# Exploring factors influencing time from dispatch to unit availability according to the transport decision in the pre-hospital setting: an exploratory study

**DOI:** 10.1186/s12873-024-00992-1

**Published:** 2024-04-29

**Authors:** Hassan Farhat, Ahmed Makhlouf, Padarath Gangaram, Kawther El Aifa, Mohamed Chaker Khenissi, Ian Howland, Cyrine Abid, Andre Jones, Ian Howard, Nicholas Castle, Loua Al Shaikh, Moncef Khadhraoui, Imed Gargouri, James Laughton, Guillaume Alinier

**Affiliations:** 1 Ambulance Service, Hamad Medical Corporation, PO Box 3050, Doha, Qatar; 2 Faculty of Sciences, University of Sfax, 3000 Sfax, Tunisia; 3 Faculty of Medicine ‘Ibn El Jazzar’, University of Sousse, 4000 Sousse, Tunisia; 4 College of Engineering, Qatar University, Doha, Qatar; 5 Faculty of Health Sciences, Durban University of Technology, PO Box 1334, Durban, 4000 South Africa; 6 Laboratory of Screening Cellular and Molecular Process, Centre of Biotechnology of Sfax, University of Sfax, Sfax, Tunisia; 7 Higher Institute of Biotechnology, University of Sfax, Sfax, Tunisia; 8 Faculty of Medicine, University of Sfax, Sfax, Tunisia; 9 University of Hertfordshire, Hatfield, UK; 10 Weill Cornell Medicine-Qatar, Doha, Qatar; 11 Northumbria University, Newcastle Upon Tyne, UK

**Keywords:** Time to event, non-conveyance, pre-hospital, EMS, ambulance response

## Abstract

**Background:**

Efficient resource distribution is important. Despite extensive research on response timings within ambulance services, nuances of time from unit dispatch to becoming available still need to be explored. This study aimed to identify the determinants of the duration between ambulance dispatch and readiness to respond to the next case according to the patients’ transport decisions.

**Methods:**

Time from ambulance dispatch to availability (TDA) analysis according to the patients’ transport decision (Transport versus Non-Transport) was conducted using R-Studio™ for a data set of 93,712 emergency calls managed by a Middle Eastern ambulance service from January to May 2023. Log-transformed Hazard Ratios (HR) were examined across diverse parameters. A Cox regression model was utilised to determine the influence of variables on TDA. Kaplan–Meier curves discerned potential variances in the time elapsed for both cohorts based on demographics and clinical indicators. A competing risk analysis assessed the probabilities of distinct outcomes occurring.

**Results:**

The median duration of elapsed TDA was 173 min for the transported patients and 73 min for those not transported. The HR unveiled Significant associations in various demographic variables. The Kaplan–Meier curves revealed variances in TDA across different nationalities and age categories. In the competing risk analysis, the ‘Not Transported’ group demonstrated a higher incidence of prolonged TDA than the ‘Transported’ group at specified time points.

**Conclusions:**

Exploring TDA offers a novel perspective on ambulance services’ efficiency. Though promising, the findings necessitate further exploration across diverse settings, ensuring broader applicability. Future research should consider a comprehensive range of variables to fully harness the utility of this period as a metric for healthcare excellence.

**Supplementary Information:**

The online version contains supplementary material available at 10.1186/s12873-024-00992-1.

## Introduction

Although referred to as emergency medical services (EMS), the rapid response of ambulance services to critical situations often stands as a defining factor between positive and negative health outcomes for individuals requiring emergency care [1]. Consequently, the dynamics of ambulance availability have come under increasing scrutiny [2, 3]. While multiple elements influence this availability—ranging from socio-demographic shifts to geographical constraints—a vital component often overlooked in the discourse is the decision-making process surrounding patient conveyance and its consequential effects on the time to handover at a medical facility where definitive care can be provided [4].

Over the last three decades, a notable surge in technological and algorithmic developments has reshaped our approach to ambulance dispatch [5–7]. With the evolution of real-time data analytics and more sophisticated mathematical models, EMS systems worldwide have strived for real-time optimisation [8, 9]. Nevertheless, a substantial gap exists in integrating patient conveyance decisions within a framework analysing time-to-event data.

With its rapid demographic metamorphosis, the Middle East, especially in countries like Qatar, necessitates an individualised approach to EMS decision-making regarding patient care and operations in general. The diverse population has varying expectations from an ambulance service and can present challenges to paramedics [10]. The uniqueness of Qatar’s health landscape and broader regional trends accentuate the need for a more intricate understanding of EMS logistics, especially with ambulance dispatch and its associated availability.

This study was prompted by the absence of an integrated exploration of time from dispatch to ambulance availability according to the transport decision in the current literature. Such an inquiry will yield novel insights with significant implications for refining EMS operational strategies.

We hypothesise that a comprehensive analysis considering conveyance decisions with time-to-event metrics will elucidate novel determinants critical to the efficacy of ambulance availability.

This study seeks to bridge the identified gap in EMS research, combining the nuances of time-to-event analysis with the practicalities of conveyance decisions. We aim to discern the essential factors shaping the duration of a case, from dispatch to completion, as they impact ambulance readiness to respond to the next case according to the patient transport decision in a Middle Eastern EMS context. By doing so, we expect to provide insights that can guide future EMS strategies, ensuring optimal resource allocation and enhanced patient care.

## Methods

### Research framework and context

A retrospective evaluation of 93,712 emergency calls managed by Hamad Medical Corporation Ambulance Service (HMCAS) was conducted from 1st January to 31st May 2023. The dataset was derived from the digital archives of HMCAS, which is managed by its Business Intelligence (BI) department. This investigation aligns with the tenets of the Strengthening the Reporting of Observational Studies in Epidemiology (STROBE) framework for cohort evaluations with ethical approval from the HMC Medical Research Center (MRC-01–22-264). All statistical analyses were performed using an R programming environment.

### Setting

In Qatar, a Middle Eastern nation with a multicultural demographic comprising mostly South Asians and Arabs, HMCAS is the governmental EMS provider. Catering for all residents and visitors, it provides emergency and non-emergency medical transport services within and beyond national boundaries [11, 12]. HMCAS ensures pre-hospital medical services for 3,118,000 citizens and residents in Qatar [13]. Furthermore, its ambulances are staffed by Ambulance Paramedics, who can perform medical assessments, determine provisional diagnoses, interpret electrocardiogram readings, and administer treatment and life-saving interventions such as supraglottic airway management and intravenous therapy. Critical Care Paramedics have a more advanced scope of practice and can perform more complex procedures, such as endotracheal intubation [14, 15].HMCAS promotes patient hospital conveyance, refraining from clinician-driven non-conveyance and emphasising patient or next-of-kin-endorsed transportation decisions [14, 15]. The reason for this is to avoid potential risks associated with under-triaging cases, particularly in elderly patients who may sometimes present with an ambiguous clinical presentation that conceals serious health issues.Upon receiving a call for service (CFS) via 999, the Emergency Medical Dispatcher (EMD) from the National Command Center (NCC) uses the ‘ProQa™’ software within the Medical Priority Dispatch System (MPDS) to rapidly dispatch the nearest ambulance available, ensuring timely medical intervention and subsequent patient transfer to a suitable healthcare facility [16].

### Participants

The investigative scope included all 999 emergency interactions between January and May 2023, that triggered the deployment of an emergency response unit, during which on-site patient assessment was undertaken, culminating in patient transport to a medical facility or a patient’s decision to forgo such transfer. The scope explicitly omitted 1) cases with fatalities, given their non-contributive nature to further clinical care, and 2) emergency calls from healthcare facilities.

### Variables and data analysis

After data cleaning, 88,479 cases were retained for the analysis, including 67,285 (46.04%) transported and 21,194 (23.95%) non-transport cases (Appendix 1).

### The time variable

The time variable in this study is denoted as ‘Time from ambulance Dispatch until Available’ (TDA), quantifying the duration from receiving a 999 emergency call to the point when the ambulance has been restocked, if necessary, and is ready for dispatch to another emergency call. This duration was measured in minutes.

### The event Indicator

The event indicator signals the occurrence of the event of interest. In this context, transport decisions serve as the variables of interest, reflecting whether a patient was transported and the unit became available for another dispatch or whether the patient decided not to be transported, signed the refusal form, and the ambulance became available on scene. Transport decisions were employed as the event indicators under our dataset’s ‘Handover’ variable.

### Censoring variable

Censoring is relevant when the event of interest is not observed. In this study, the contrary transport decision under the ‘Handover’ variable was treated as the censoring variable, indicating situations where the patient refused transport and the unit became available on scene, or the patient was transported, handed over to a receiving hospital, and the ambulance became available. As delineated in a preceding publication, patient non-transport in HMCAS exclusively arises due to patient decision [14].

The prediction variables:

These are the variables that might influence the TDA and are defined by:

- Demographic predictors: Gender, nationality categories, age categories, and weight categories.
- Comorbidities: Asthma, hypertension, diabetes mellitus (DM), coronary artery diseases (CAD), cerebral vascular accidents (CVA), Cardiopulmonary obstructive diseases (COPD), unknown, and others.
- Other variables: CFS owner, which refers to call-for-service (case reference number) recorded by 999 and may be attributed to HMCAS or the Ministry of Interior (Police or Civil Defence). In addition to call-taking ProQA™ protocol, response-to-scene priority, response unit type, hour of the day when the 999 calls were received, the provisional diagnoses determined by the paramedics after initial assessment of the patient, transport destination, and the exact destination at the hospital were included. Other time variables were considered as covariates, such as the time it took to identify the closest unit to the 999 emergency caller location, the time the ambulance took to reach the patient’s location, and the time spent by the paramedics on scene.

## Data analysis

### Assessment of multicollinearity

To ensure the assumptions of the Cox Proportional Hazard model were met, potential multicollinearity amongst the independent variables was evaluated. A linear model was constructed to predict the time from ambulance dispatch until availability based on variables such as CFS owner, unit type, and gender. The Generalised Variance Inflation Factor (GVIF) was calculated for each predictor to quantify the severity of multicollinearity. Variables with GVIF values greater than 10 were considered to have high multicollinearity.

## Survival analysis

### Data preparation

Variables were prepared for the Cox model analysis after assessing multicollinearity. Composite variables were produced to facilitate streamlined interpretation. Concurrently, relevant categorical variables were converted to factor variables.

### TDA Analysis

A TDA analysis was undertaken using the Cox Proportional Hazards model. Within this model, the influence of various covariates on the hazard was measured by hazard ratios (HR). The TDA was defined as the outcome variable, while this event was identified based on whether the transportation of a patient had occurred. A diverse array of predictors, which included demographic variables, details of dispatch, and pre-existing comorbidities, was incorporated into the model.

Then, potential effect modification was assessed. An interaction between gender and age categories was introduced into a separate Cox model to determine any possible effect modification.

In addition, a rigorous evaluation of the model was carried out. Analysis of Variance (ANOVA) tests were employed to determine the significance of each predictor within the model. Simultaneously, the validity of the proportional hazards assumption was verified using the Schoenfeld residual plot.

A Kaplan–Meier Survival Analysis was conducted. Kaplan–Meier survival curves were plotted to visually represent unadjusted survival probabilities over time. The timespan from ambulance dispatch to availability was selected as the primary endpoint, with categorisation based on the transport status of patients. Kaplan–Meier curves were further stratified, highlighting distinctions such as gender, top three nationality categories, age cohorts, weight classifications, and dominant comorbidities whilst excluding categories like’ ‘None’, ‘Unknown’, and’ ‘Others’ visual representation of HR, their associated confidence intervals, and the statistical significance of each predictor relative to the primary outcome [17].

Forest plots were generated to portray HR and their respective confidence intervals. This visual aid reinforced the representation of a Cox model for each variable.

Lastly, a competing risk analysis was executed. This approach allowed for concurrently evaluating both outcomes — patient ‘Transported’ and ‘Not Transported’, offering a comprehensive understanding of patient trajectories. Competing risk analysis is a statistical approach used to assess the probability of different types of events, taking into account that the occurrence of one event may hinder the occurrence of another. This method is beneficial as it provides more accurate and nuanced insights into event probabilities, particularly in clinical studies where patients may experience different events, compared to the Kaplan–Meier method, which can overestimate the cumulative incidence of events in the presence of competing risks [17]. The status variable was codified such that events denoted as a patient ‘Transported’ were labelled as 1, patient ‘Not Transported’ events as 2, and non-occurrences of any event as 0. Subsequently, the Cumulative Incidence Function (CIF) for the competing risks associated with these events was computed. For visualisation, the CIF was plotted, contrasting the risks of ‘Transported’ versus ‘Not Transported’ over time, with blue and red hues, respectively. Furthermore, the median time to the event where patients were not transported was determined, and this duration, derived from a binary encoding of the ‘Not Transported’ event, was showcased alongside its 95% confidence interval for clarity in the interpretation. In the analysis, a *p*-value of less than 0.05 was considered statistically significant.

## Results

**Fig. 1:**
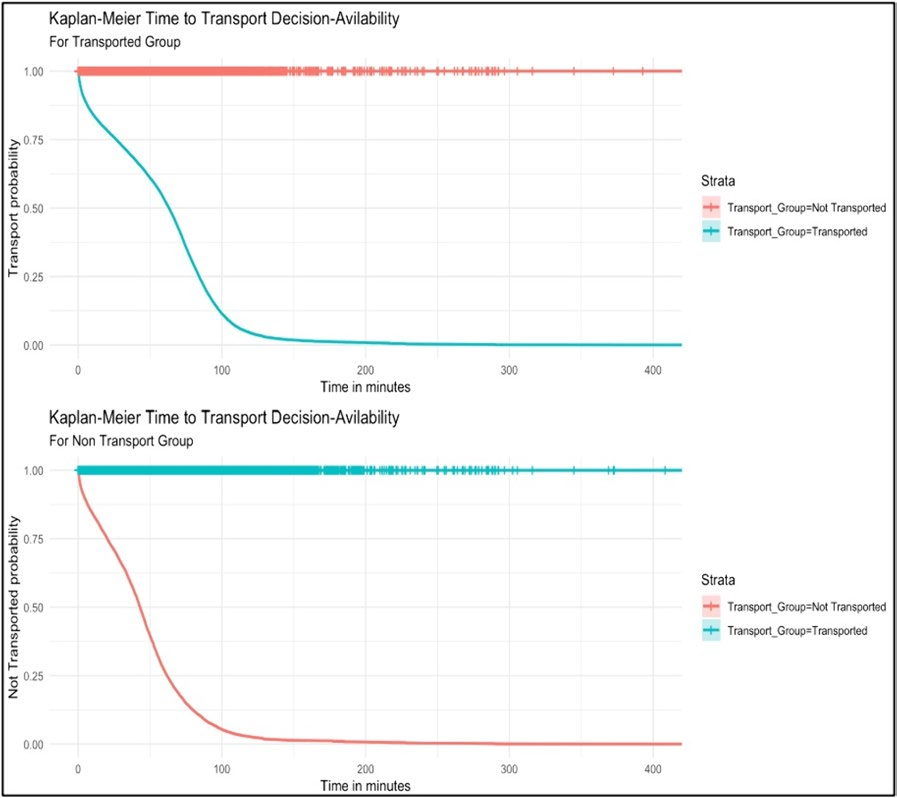
Overall Population Kaplan–Meier plot

The results showed that the median TDA was 173 min for transported patients, with a 95% confidence interval ranging from 158 to 177 min. For patients who were not transported, the median TDA was significantly shorter at 70 min, with a 95% confidence interval between 69 and 70 min. (Fig. 1).

When assessing multicollinearity amongst predictor variables using the GVIF elevated GVIF values were identified in several variables (Appendix 2 and 3). The variables with GVIF close to 1 suggested minimal multicollinearity concerns.

The HRs of both groups were determined and are shown in Tables 1 and 2.

**Table 1:** Hazard Ratio analysis results based on demographic information and ambulance dispatch metrics

	**Transported**		**Not Transported**			
Characteristic	**log(HR)**^***1***^	**95% CI**^***1***^	***p*-value**	**log(HR)**^***1***^	**95% CI**^***1***^	***p*-value**
Owner_UnitType (CFS owner&Unit type)						
EMS_Alpha	—	—		—	—	
EMS_Bravo	0.02	-0.07, 0.11	0.7	0.43	0.36, 0.51	< 0.001
EMS_Charlie	0.43	0.39, 0.47	< 0.001	0.19	0.09, 0.28	< 0.001
EMS_Delta	0.48	0.43, 0.53	< 0.001	-0.01	-0.11, 0.09	0.9
EMS_HAZMAT	0.46	0.40, 0.52	< 0.001	0.22	0.09, 0.35	< 0.001
EMS_LF	0.33	0.18, 0.47	< 0.001	-0.35	-0.82, 0.11	0.14
EMS_MIR	0.5	0.43, 0.56	< 0.001	0.38	0.25, 0.50	< 0.001
EMS_Missing	-10	-659, 639	> 0.9	0.69	-1.3, 2.7	0.5
EMS_Other	0.47	0.41, 0.53	< 0.001	0.11	-0.02, 0.23	0.1
No call taking_Missing	0.12	0.06, 0.18	< 0.001	0.44	0.32, 0.55	< 0.001
Other_Alpha	-0.25	-0.31, -0.20	< 0.001	-0.1	-0.19, 0.00	0.056
Other_Bravo	-0.05	-0.79, 0.70	> 0.9	-0.16	-1.3, 0.98	0.8
Other_Charlie	0.13	0.06, 0.20	< 0.001	0.35	0.21, 0.49	< 0.001
Other_Delta	0.09	0.02, 0.16	0.008	0.14	0.03, 0.26	0.017
Other_HAZMAT	0.36	0.16, 0.56	< 0.001	0.1	-0.24, 0.45	0.6
Other_LF	0.32	0.20, 0.44	< 0.001	-0.25	-0.54, 0.04	0.089
Other_MIR	-0.08	-0.26, 0.09	0.4	-0.38	-0.71, -0.06	0.021
Other_Missing	-8.7	-195, 178	> 0.9	3.3	1.9, 4.6	< 0.001
Other_Other	0.08	0.00, 0.17	0.049	-0.57	-0.77, -0.38	< 0.001
DispatchType						
Zulu (Z)	—	—	—	—	—	
Yankee (Y)	-0.04	-0.07, 0.00	0.025	0.07	0.00, 0.13	0.035
Xray (X)	-0.02	-0.07, 0.02	0.3	1.4	-0.55, 3.4	0.2
Tango (T)	0.35	-0.06, 0.76	0.1	1.1	0.64, 1.6	< 0.001
UncompletedProQA	-0.01	-0.25, 0.23	> 0.9	0.26	0.17, 0.34	< 0.001
Not in use	-9.8	-455, 435	> 0.9	3.6	2.6, 4.5	< 0.001
Priority To Scene						
P1	—	—		—	—	
P2	-0.01	-0.04, 0.01	0.3	0	-0.04, 0.04	> 0.9
Missing	-0.25	-0.36, -0.13	< 0.001	0.74	0.63, 0.85	< 0.001
Region						
Rural	—	—		—	—	
Unknown	0.32	-0.07, 0.71	0.11	-1.3	-2.7, 0.12	0.074
Urban	0.03	0.01, 0.06	0.002	-0.03	-0.08, 0.01	0.12
Location Type						
Airport	—	—		—	—	
Beach/Sea/Ocean	0.37	0.24, 0.50	< 0.001	-0.72	-0.94, -0.49	< 0.001
Farm	0.28	0.14, 0.43	< 0.001	-1.1	-1.4, -0.72	< 0.001
Home	0.44	0.39, 0.49	< 0.001	-0.86	-0.92, -0.81	< 0.001
Industrial Area	0.55	0.48, 0.62	< 0.001	-1.8	-1.9, -1.6	< 0.001
Other	0.22	0.16, 0.28	< 0.001	-0.65	-0.74, -0.57	< 0.001
Public Area	0.31	0.25, 0.38	< 0.001	-0.8	-0.89, -0.71	< 0.001
Recreation (Sport)	0.42	0.29, 0.56	< 0.001	-0.73	-1.0, -0.46	< 0.001
School	0.47	0.40, 0.55	< 0.001	-0.83	-0.95, -0.71	< 0.001
Street (Road)	0.49	0.44, 0.55	< 0.001	-0.68	-0.74, -0.61	< 0.001
Work	0.56	0.50, 0.62	< 0.001	-1.2	-1.3, -1.1	< 0.001
Missing	0.23	0.16, 0.30	< 0.001	-0.71	-0.81, -0.61	< 0.001
Gender						
Female	—	—		—	—	
Male	0.02	0.01, 0.04	0.009	-0.2	-0.29, -0.10	< 0.001
Missing	0.09	-0.33, 0.50	0.7	-0.86	-2.3, 0.53	0.2
Nationality categories						
East Asia & Pacific	—	—		—	—	
Europe & Central Asia	-0.23	-0.29, -0.16	< 0.001	0.04	-0.05, 0.13	0.4
GCC Other	-0.1	-0.15, -0.04	< 0.001	0.12	0.02, 0.21	0.014
Latin America & Caribbean	-0.02	-0.23, 0.19	0.9	-0.04	-0.34, 0.26	0.8
MENA	-0.08	-0.12, -0.04	< 0.001	0.35	0.28, 0.42	< 0.001
Missing	-0.14	-0.21, -0.07	< 0.001	-0.07	-0.20, 0.07	0.3
North America	-0.04	-0.17, 0.09	0.6	0.03	-0.15, 0.21	0.8
Other	-0.06	-0.13, 0.01	0.083	0.06	-0.05, 0.17	0.3
Qatar	-0.17	-0.21, -0.13	< 0.001	0.48	0.40, 0.55	< 0.001
South Asia	0.02	-0.01, 0.06	0.2	-0.02	-0.09, 0.05	0.5
Sub-Saharan Africa	0.02	-0.02, 0.06	0.3	0.11	0.04, 0.19	0.005
Age categories (years)						
14 ≤ Age < 29	—	—		—	—	
29 ≤ Age < 44	0	-0.02, 0.02	> 0.9	0.02	-0.01, 0.06	0.2
44 ≤ Age < 59	0.04	0.02, 0.07	< 0.001	-0.07	-0.12, -0.02	0.007
59 ≤ Age < 75	-0.01	-0.04, 0.03	0.7	0.04	-0.03, 0.10	0.3
75 ≤ Age < 90	-0.07	-0.12, -0.02	0.004	0.08	-0.01, 0.17	0.072
Age < 14	0.11	0.06, 0.17	< 0.001	0.01	-0.09, 0.11	0.8
Age ≥ 90	-0.18	-0.28, -0.07	0.001	0.06	-0.14, 0.26	0.5
Missing	0.02	-0.15, 0.18	0.8	-16	-1,016, 984	> 0.9
Weight categories (kg)						
45 ≤ Weight < 70	—	—		—	—	
70 ≤ Weight < 95	0	-0.02, 0.02	> 0.9	-0.02	-0.05, 0.01	0.2
95 ≤ Weight < 120	0.01	-0.03, 0.04	0.6	-0.18	-0.25, -0.11	< 0.001
Weight < 45	-0.05	-0.10, 0.01	0.1	-0.06	-0.16, 0.04	0.2
Weight ≥ 120	-0.1	-0.18, -0.02	0.012	-0.37	-0.53, -0.21	< 0.001
Missing	0.49	0.20, 0.78	< 0.001	-0.96	-1.9, 0.03	0.058

^*1*^ *HR* Hazard Ratio, *CI* Confidence Interval

**Table 2:** Hazard Ratio Analysis ProQA protocols, Provisional diagnoses and Comorbidities

	**Transported**			**Not Transported**		
Name	**log(HR)**^***1***^	**95% CI**^***1***^	***p*-value**	**log(HR)**^***1***^	**95% CI**^***1***^	***p*-value**
ProtocolName						
Abdominal pain (P1)	—	—	—	—	—	—
Abnormal Behaviour (P25)	0.12	0.03, 0.22	0.013	-0.14	-0.31, 0.04	0.13
Allergies_Envenomations (P2)	-0.02	-0.14, 0.10	0.8	0.27	0.05, 0.49	0.016
Animal bites (P3)	0.27	-0.01, 0.54	0.056	-16	-2,086, 2,054	> 0.9
Assault (P4)	-0.05	-0.15, 0.04	0.3	0.89	0.74, 1.0	< 0.001
Back Pain (P5)	0	-0.07, 0.07	> 0.9	-0.27	-0.42, -0.13	< 0.001
Breathing problems (P6)	-0.04	-0.09, 0.02	0.2	0.18	0.08, 0.28	< 0.001
Burns (P7)	0.06	-0.08, 0.20	0.4	-0.08	-0.34, 0.18	0.6
Cardiac arrest (P9)	0.03	-0.09, 0.15	0.6	0.2	-0.16, 0.56	0.3
CBRN (P8)	-0.7	-0.96, -0.43	< 0.001	0.37	0.02, 0.71	0.04
Chest pain (P10)	0.04	-0.02, 0.10	0.2	0.13	0.01, 0.24	0.028
Choking (P11)	0.02	-0.16, 0.21	0.8	0.63	0.38, 0.88	< 0.001
Convulsions (P12)	0.02	-0.07, 0.10	0.7	0.42	0.25, 0.59	< 0.001
Criminal Incidents (None_EMS)	-0.13	-0.38, 0.12	0.3	0.49	0.15, 0.82	0.005
CVA_TIA (P28)	0.04	-0.10, 0.18	0.5	0.31	0.01, 0.60	0.044
Diabetic Problems (P13)	-0.18	-0.29, -0.06	0.003	0.38	0.22, 0.54	< 0.001
Drowning related (P14)	0.61	-0.13, 1.4	0.11			
Electrocution (P15)	-0.23	-0.69, 0.23	0.3	0.87	0.32, 1.4	0.002
Entrapments (P22)	-0.58	-0.71, -0.44	< 0.001	0.68	0.41, 0.95	< 0.001
Eye problems (P16)	0.04	-0.14, 0.23	0.6	-0.12	-0.42, 0.18	0.4
Falls (P17)	0.13	0.07, 0.19	< 0.001	-0.15	-0.29, -0.01	0.037
Fire (None_EMS)	-0.14	-0.80, 0.52	0.7	0.95	0.32, 1.6	0.003
Headache (P18)	-0.02	-0.11, 0.07	0.6	0.19	0.05, 0.33	0.008
Heart Problems (P19)	-0.19	-0.28, -0.09	< 0.001	0.31	0.15, 0.47	< 0.001
Hemorrhage (P21)	0.16	0.07, 0.24	< 0.001	-0.08	-0.26, 0.11	0.4
Maritime (None_EMS)	0.96	-1.0, 2.9	0.3			
Miscarriage_Pregnancy (P24)	0.1	0.00, 0.19	0.039	-0.41	-0.76, -0.06	0.023
Others	-0.03	-0.15, 0.08	0.6	-3.1	-3.9, -2.2	< 0.001
Penetrating Trauma (P27)	-0.74	-0.98, -0.50	< 0.001	-0.94	-1.6, -0.33	0.003
Poisoning (ingestion) (P23)	-0.05	-0.23, 0.12	0.6	0.07	-0.25, 0.38	0.7
Rescue (None_EMS)	1.1	0.29, 1.9	0.007			
Road Traffic Accidents (P29)	0.28	0.21, 0.35	< 0.001	0.55	0.42, 0.69	< 0.001
Sick person (P26)	-0.04	-0.09, 0.02	0.2	0.12	0.03, 0.22	0.01
Traumatic Injuries (P30)	0.13	0.06, 0.20	< 0.001	0.04	-0.10, 0.17	0.6
Unconscious (P31)	0.02	-0.04, 0.08	0.5	0.13	0.02, 0.24	0.021
Unknown Problem (P32)	0.19	0.04, 0.34	0.013	-0.01	-0.28, 0.26	> 0.9
ProvisonalDiagnoses_CAT						
Acute trauma	—	—		—	—	
Allergy/Anaphylaxis	0.11	0.01, 0.20	0.026	0.04	-0.16, 0.25	0.7
Asthma	-0.27	-0.35, -0.19	< 0.001	0.45	0.30, 0.60	< 0.001
Burn	-0.08	-0.19, 0.03	0.2	0.25	0.04, 0.46	0.018
Cardiac Arrest	-0.04	-0.14, 0.05	0.4	-14	-310, 281	> 0.9
Cardiovas_ACS	0.05	-0.01, 0.11	0.089	-0.35	-0.54, -0.17	< 0.001
Cardiovas_Other	0.24	-1.1, 1.6	0.7			
Cardiovas_SupraVentricular	0.18	0.07, 0.28	< 0.001	-0.75	-1.1, -0.36	< 0.001
Cardiovas_Ventricular	-0.18	-0.64, 0.29	0.5	-1.4	-3.4, 0.52	0.15
Chronic Condition	-0.01	-0.07, 0.05	0.7	0.09	-0.04, 0.23	0.2
COPD	-0.46	-0.70, -0.22	< 0.001	-0.33	-1.0, 0.35	0.3
COVID19_Related	-0.16	-0.24, -0.07	< 0.001	0.01	-0.19, 0.21	> 0.9
Croup/Epiglottitis	0.12	-0.07, 0.31	0.2	-1.6	-2.8, -0.51	0.005
CVA/TIA	0.12	0.02, 0.23	0.021	-1.1	-1.7, -0.59	< 0.001
Electrocution	0.32	-0.22, 0.87	0.2	0.51	-0.64, 1.7	0.4
Endocrinology_Other	0.51	-0.30, 1.3	0.2			
Envenomation	-0.01	-0.37, 0.36	> 0.9	0.14	-0.74, 1.0	0.7
FBAO	-0.09	-0.34, 0.16	0.5	0.44	-0.09, 0.97	0.11
Fever	-0.15	-0.19, -0.11	< 0.001	0.57	0.48, 0.65	< 0.001
GI	-0.03	-0.07, 0.02	0.2	0.16	0.06, 0.26	0.001
GU	0.21	0.11, 0.31	< 0.001	-0.67	-1.1, -0.28	< 0.001
Heat_related	-0.15	-0.42, 0.13	0.3	-0.09	-0.90, 0.71	0.8
Hemothorax	0.55	0.01, 1.1	0.047			
Hyperglycemia	-0.08	-0.18, 0.02	0.11	0.47	0.28, 0.65	< 0.001
Hypertension	0.09	0.00, 0.18	0.046	-0.2	-0.48, 0.08	0.2
Hypoglycemia	-0.53	-0.67, -0.38	< 0.001	1.3	1.1, 1.4	< 0.001
Low Acuity Problem_Medical	-0.25	-0.29, -0.22	< 0.001	0.85	0.78, 0.92	< 0.001
Low Acuity Problem_Trauma	-0.05	-0.08, -0.02	0.004	0.5	0.42, 0.57	< 0.001
Near drowining	-0.17	-1.6, 1.2	0.8	1.1	-0.89, 3.1	0.3
Neurology_Other	-0.27	-0.32, -0.21	< 0.001	0.73	0.64, 0.83	< 0.001
OBS_GYN	0.17	0.10, 0.25	< 0.001	-1.7	-2.0, -1.3	< 0.001
Other	0	-0.05, 0.05	> 0.9	-0.25	-0.39, -0.11	< 0.001
Pneumothorax	-0.36	-1.2, 0.52	0.4			
Respiratory_Infection	-0.11	-0.16, -0.05	< 0.001	0.4	0.29, 0.51	< 0.001
Respiratory_Other	-0.31	-0.40, -0.21	< 0.001	0.95	0.81, 1.1	< 0.001
Seizure	0.11	0.03, 0.18	0.007	-0.72	-0.95, -0.50	< 0.001
Shock	0.26	0.02, 0.51	0.034	-15	-1,847, 1,816	> 0.9
Syncope	0	-0.09, 0.09	> 0.9	0.59	0.43, 0.76	< 0.001
Toxicology	-0.07	-0.17, 0.03	0.2	0.24	0.02, 0.46	0.032
Co-morbidities						
Asthma	-0.02	-0.06, 0.03	0.5	-0.02	-0.11, 0.06	0.6
CAD	-0.02	-0.06, 0.02	0.4	-0.01	-0.09, 0.07	0.8
COPD	0.12	0.00, 0.25	0.059	0.04	-0.22, 0.30	0.8
CVA	0.06	-0.01, 0.13	0.07	-0.42	-0.59, -0.25	< 0.001
Seizure	0.09	0.02, 0.16	0.016	0.05	-0.12, 0.22	0.6
DM	0.05	0.02, 0.08	< 0.001	0.01	-0.04, 0.06	0.7
Hypertension	0.05	0.02, 0.08	0.001	-0.1	-0.15, -0.04	< 0.001
Surgeries	0.11	0.06, 0.15	< 0.001	-0.27	-0.38, -0.17	< 0.001
CurrentlyPregnant	0.04	-0.02, 0.10	0.2	-0.3	-0.42, -0.17	< 0.001

^*1*^ *HR* Hazard Ratio, *CI* Confidence Interval

**Fig. 2:**
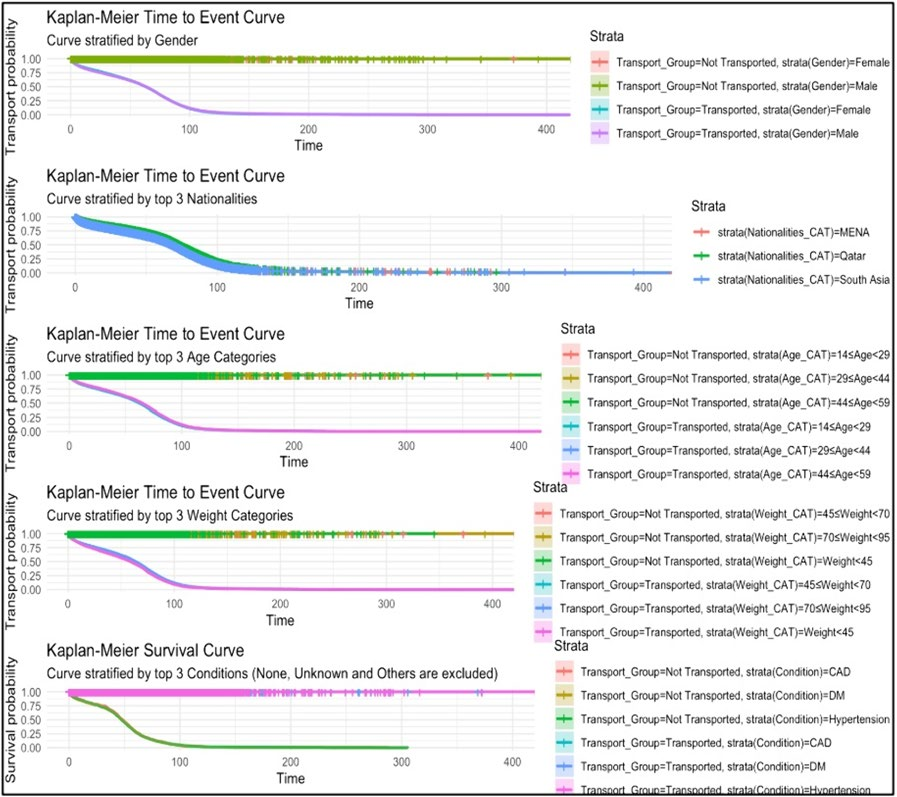
Kaplan–Meier plots for the stratification variables of the transported group

**Fig. 3:**
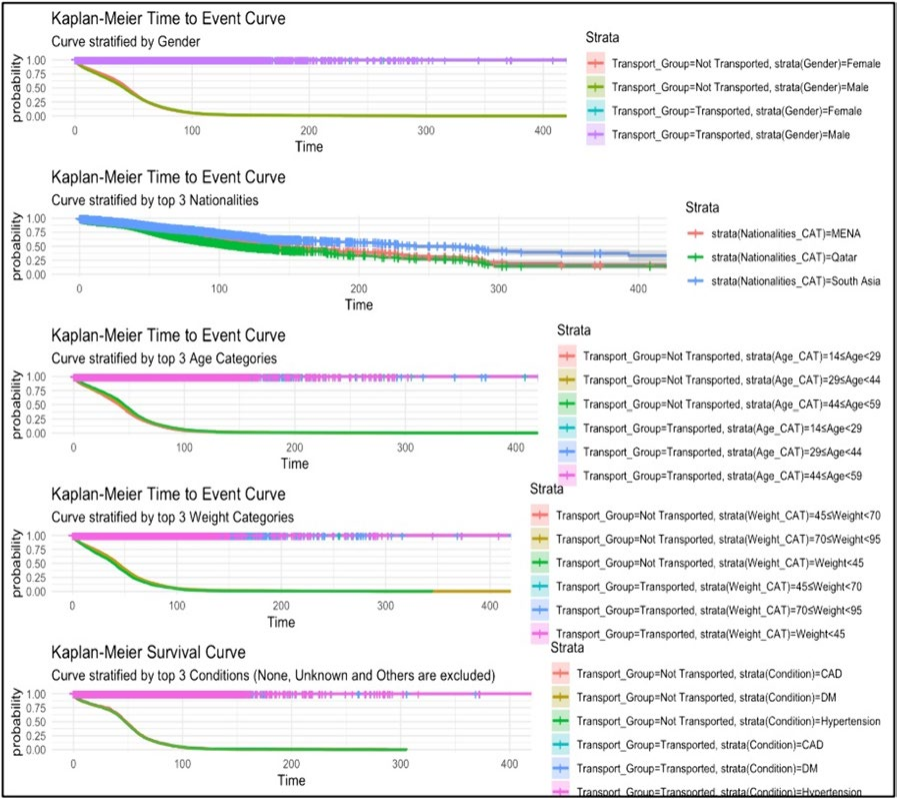
Kaplan–Meier plots for the stratification variables of non-transported group

Moreover, within the demographic variables, the log HR and *p*-value of the ‘Europe and Central Asia’ transported group (HR = -0.023; *p* < 0.001) suggest a lower likelihood the TDA to be shorter than the other nationatlities. For the other GCC Transported Group, excluding Qatar, indicates a lower likelihood the TDA to be shorter. Similarly, the MENA transported group, with an HR of -0.08 and a *p*-value < 0.001, signifies a lower likelihood the TDA to be shorter. However, the MENA not transported group, with an HR of 0.35 and a *p*-value < 0.001, indicates a higher likelihood of the TDA. For the Qatari transported group, the HR of -0.17 and a *p*-value < 0.001 denotes a lower likelihood of the TDA to be shorter.This was also confirmed by Figs. 2 and 3.

Age and certain health conditions also played a role in TDA variations. Younger patients (under 14 years old) and those with specific comorbidities like hypertension and Diabetes Mellitus experienced longer TDAs. In contrast, patients aged between 75 and 90 years generally had shorter TDAs.

For the transported group, COPD had HR = 0.12, suggesting a potential increase due to the borderline *p*-value 0.05. In the not-transported group, the maximal HR was attributed to seizures (HR = 0.05). CVA had the lowest HR in this cohort, demonstrating an HR = -0.42 and a highly significant *p*-value of < 0.001. Furthermore, a substantial elevation was observed for patients with hypertension and DM in the transported group, contrasted by a significant reduction in risk for hypertension within This.

**Fig. 4:**
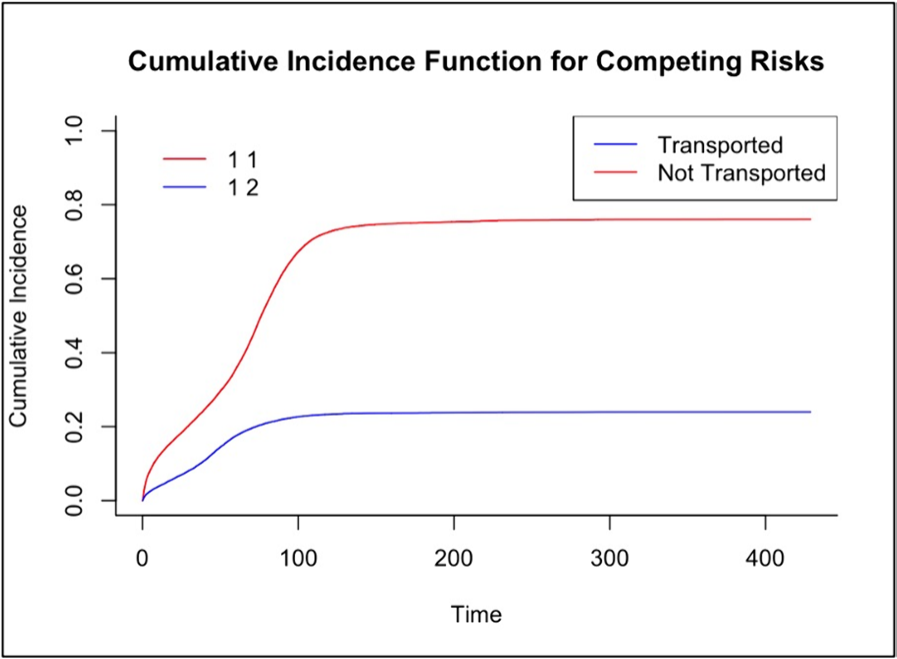
Competing Risk Analysis Results

The Cox regression model assessed the influence of various factors on TDA (Appendix 4 and 5), revealing both positive and negative associations. Kaplan–Meier curves further highlighted differences in TDA across various patient groups, informing us about the likelihood of different outcomes based on patient characteristics. Finally, competing risk analysis (Fig. 4) showed that non-transported patients tended to wait longer before deciding than those transported.

Provisional diagnoses and EMS protocols were additional factors affecting TDA. For example, patients with conditions like “Hemothorax” or under protocols like “Rescue” for the transported group showed longer TDAs, indicating a need for more time to manage these cases.

These findings highlight the complexity of factors influencing ambulance readiness and suggest areas for future policy and operational improvements to optimise EMS services.

## Discussion

In EMS research, time from dispatch to the ambulance becoming available is a notably under-researched metric despite its potential implications for service provision efficiency and patient outcomes. Historically overshadowed by other parameters, understanding this metric’s complexities can offer insights into healthcare system operations, shedding light on areas of inefficiency or excellence [18, 19]. This study ventures into an often-neglected domain, proposing TDA as a pioneering tool to build for Quality Improvement (QI) studies. Its relevance spans diverse healthcare contexts, making it universally applicable and paramount for global collaborations and benchmarking [20–22]. As a measurable metric influenced by multidimensional variables—infrastructural, policy-driven, or human resources—it offers a tangible avenue for targeted interventions, ultimately aiming to enhance patient care. Drawing analogies from other sectors where time metrics dictate operational efficiencies, our exploration of the TDA according to the transport decision is to respond to another case, and this aligns with global healthcare standards of excellence. This novel QI tool, as this study demonstrated, not only fills a gap in the literature but has the potential to redefine facets of healthcare quality, paving the way for future research and policy-making by including this type of time-to-event (and its constituent determinants) as a quality indicator in pre-hospital care.

The study findings offered a comprehensive understanding of the determinants influencing the time an ambulance becomes available. The median TDA duration was 173 min for the transported group compared to 70 min for the not-transported group. Similar studies have reported various durations attributable to regional differences, institutional practices, or population demographics [23, 24]. Further, the significant difference in TDA may also reflect varying levels of clinical need between the sub-groups of patients being transported or not transported. It implies that transported patients, potentially with more severe or complex conditions, require longer on-scene times for stabilisation or treatment before transportation, enhancing patient-centric care by addressing the specific needs of individual patients. Recognising these disparities is crucial to building robust QI initiatives, especially when benchmarking against global best practices. In addition, it is essential to consider the competencies of healthcare workers as a potential influence on the TDA. The variability in TDA between patient groups being transported may not solely reflect the clinical needs of the patients but also the decision-making skills, efficiency, and clinical expertise of the EMS personnel involved. Competent and quick decision-making, rooted in extensive experience and training, can significantly impact the time required for on-scene stabilisation and treatment, thereby affecting TDA. This perspective suggests that looking at and then enhancing, if needed, the training and skills of EMS staff could be as crucial as addressing patient-centric factors in reducing TDA and optimising pre-hospital care delivery. This also demonstrates the importance of continuous professional development and support for EMS personnel as part of broader QI initiatives.

The detected elevated GVIF values, particularly in the CFS owner and unit type variables, mirror concerns raised in the literature about the potential collinearity [25, 26] addressed by creating new variables.

An examination of log-transformed HRs highlighted strong associations across ‘nationalities, age, and other categories reminiscent of findings from similar studies [14, 27]. Understanding such relationships aids in stratifying risk and tailoring patient-centric interventions. For instance, differences in HRs observed within various subcategories and demographic variables hold substantial implications for clinical EMS practice. Elevated HRs in variables such as nationality categories, age categories, and ProQA™ protocols suggest that these factors may significantly influence the likelihood of transport decision events. The differences in HRs between transported and not transported groups, particularly among different nationality categories and age groups, necessitate a nuanced understanding of demographic-specific risks. For instance, the statistically significant lower HR in the Europe and Central Asia, other GCC, and MENA transported groups compared to the East Asia and Pacific group illustrates the demographic disparities in transport likelihoods, thereby the TDA. This, associated with contrasting HRs observed within Qatari groups, highlights the need for individualised risk assessments and tailored intervention strategies based on demographic attributes.

Furthermore, the distinctive HR patterns within comorbidities and provisional diagnoses, such as the elevated risk observed in COPD within the transported cohort and the significant HRs in individuals with a history of surgeries in both groups, require enhanced medical vigilance and specialised care strategies for these groups. The significant elevation in risk observed for patients with hypertension and DM in the transported group and the contrasting reduction in risk within the not-transported group exemplify the necessity for targeted medical interventions and transport decisions based on specific comorbidities and diagnoses. Additionally, the correlation between the provisional diagnosis of ‘Hemothorax” and higher HRs in the transported group, along with the prominent HRs in specific call-taking ProQA™ protocols like the “Rescue” and “Assault” protocols, highlights the relevance of protocol-specific approaches in managing and mitigating risks associated with different medical emergencies and trauma cases. The significant variations in HRs among different provisional diagnoses and protocols emphasise the imperative of a comprehensive and integrated approach in EMS practice that accounts for the intersecting factors affecting transport decision events. These observations collectively advocate for a refined and multifactorial approach in EMS clinical practice, focusing on demographic-specific, comorbidity-related, and protocol-oriented strategies to optimise the unit availability transport decisions and enhance patient care outcomes. The evident disparities and variations in HRs across different groups and categories necessitate the continual refinement of EMS protocols and interventions to effectively address the diverse and evolving needs of the varied patient populations.

The Cox regression model analysis findings reaffirm the importance of factoring in diverse variables when assessing TDA. Some studies have highlighted the significance of time-related variables, making our results lack influence on the time when the 999 emergency call was received an interesting deviation.

Kaplan–Meier curve findings underscore the nuanced disparities in TDA based on demographics and clinical variables. While some studies confirm the gender-based uniformity in transport probability rates [28, 29], the observed variability across nationality categories, especially between the Qatari and South Asian cohorts, calls for introspection, a notion explored by several researchers [30].

Furthermore, certain studies have examined the interplay between age, weight, and transport times, lending credence to our findings about pronounced time from dispatch to ambulance availability rates among specific cohorts [31].

The juxtaposition of the transported group predictor against a null model and the resultant divergent findings across tests underscore the sentiments shared in several academic publications about the importance of methodological pluralism [32, 33].

The outcome of competing risk analysis indeed resonates with extensive literature, advocating for a comprehensive perspective in interpreting health outcomes [34]. This analysis reveals a nuanced aspect of pre-hospital care decisions: while our data show shorter TDA for patients not requiring transport (70 min) compared to those who are transported (173 min), the decision-making process for not transporting patients involves careful consideration. This includes evaluating the acuteness of the medical emergency, respecting the patient’s wishes, and considering systemic factors such as potential emergency department overcrowding. While not directly increasing the measured TDA, these factors reflect the significant time and resource commitment by EMS personnel in ensuring appropriate care decisions are made on scene. Acknowledging these diversified elements is essential, offering a structured foundation for QI initiatives, thereby promoting enhanced patient outcomes and streamlined healthcare delivery systems.

Furthermore, it is pertinent to consider the role of Helicopter EMS (HEMS) in enhancing the speed of transport and overall readiness of EMS. It plays a crucial role in providing rapid transportation from the scene to healthcare facilities, significantly when ground transport may be hindered by distance, traffic, or terrain (the desert in the case of Qatar) [35, 36]. In Qatar, HEMS operates as an integral component of the system, offering an alternative means of providing rapid patient transportation that can be particularly vital in rural or hard-to-reach areas. It is imperative to acknowledge that wehre other EMS systems uses the National Advisory Committee for Aeronautics (NACA) scale for patient assessment [37, 38], the HMCAS adheres to the indications of our Clinical Practice Guidelines (CPG). The CPGs include an alternative methodology more relevant to the region’s operational healthcare operational requirements [15, 39].

In addition, restocking time also affects the TDA. Hence, a QI study was conducted in HMCAS to streamline the restocking inventory management [40].

### Limitations

In considering the results of this study, several limitations must be acknowledged. The investigation focused on a specific demographic, potentially limiting the generalisability of our findings to broader populations. The retrospective nature of the research might introduce biases such as selection and information bias. Despite thorough data collection, there were instances of missing data that might influence the results’ validity. There was also an inherent constraint tied to the available variables for analysis; other potentially significant predictors of time from dispatch to unit availability may have been overlooked, which could lead to potential residual confounding aspects. Some of these include the geographical perimeter covered by the ambulance units, whether in a rural or urban area, and the proximity of the healthcare facility to which they had to transport the patient. In future research, incorporating Geographic Information Systems data could allow for precise measurement of distances between incident locations and healthcare facilities, enabling an understanding of how these distances impact EMS response times. Moreover, external factors like regional healthcare infrastructure, policies, and cultural practices that can influence time from dispatch to the unit becoming available again were not controlled for, possibly adding unmeasured biases to the results.

Additionally, HMCAS utilises an electronic patient care record system where clinical and care records are documented using tablets. This system is designed to streamline the recording process and reduce the amount of missing data in the clinical information, which was not the case in our study. We observed considerable missing values for transported and non-transported groups, increasing the risk of incomplete information for those who refused transport without medical follow-up. Future policies should focus on recording all information completely, perhaps by integrating a feature into the system that mandates information entry in all relevant fields before a case can be closed.

## Conclusion

The analysis of time from ambulance dispatch to it becoming available to respond to the next case provides a valuable perspective on healthcare system efficiency and patient-centric care. While our findings offer essential insights, they are a starting point, warranting further exploration in diverse settings to enhance the comprehensiveness and generalisability of these observations. The potential of this metric as a novel QI tool is evident, emphasising the value of incorporating it into routine healthcare metrics. Future studies should expand on our work, considering a broader range of variables and integrating multifaceted strategies to refine and enhance the utility of time to the units becoming available as a critical metric for healthcare excellence.

### Supplementary Information

**Supplementary Material 1.**

## Data Availability

The data is available with the first author, and it is available upon reasonable request and pending approval from the Hamad Medical Corporation Medical Research Center.

## Abbreviations

ANOVA: Analysis of Variance
EMS: Emergency Medical Services
BI: Business Intelligence
GVIF: Generalised Variance Inflation Factor
CAD: Coronary Artery Diseases
HR: Hazard Ratio
CFS: Call for Service
HEMS: Helicopter Emergency Medical Services
HMCAS: Hamad Medical Corporation Ambulance Service
CIF: Cumulative Incidence Function
MPDS: Medical Priority Dispatch System
COPD: Chronic Obstructive Pulmonary Disease
NCC: National Command Center
CVA: Cerebral Vascular Accidents
QI: Quality Improvement
DM: Diabetes Mellitus
STROBE: Strengthening the Reporting of Observational Studies in Epidemiology
EMD: Emergency Medical Dispatcher
TDA: Time from Ambulance Dispatch to Availability
