# Supplementary material for: Exploring factors influencing time from dispatch to unit availability according to the transport decision in the pre-hospital setting: an exploratory study
Source: BMC Emerg Med. 2024 Apr 29;24:77. doi: 10.1186/s12873-024-00992-1 (PMC11057082; doi:10.1186/s12873-024-00992-1)
Supplement: Supplementary file 1 — Supplementary Material 1. [file 12873_2024_992_MOESM1_ESM.pdf]

Appendixes

Appendix 1: The Study Flowchart

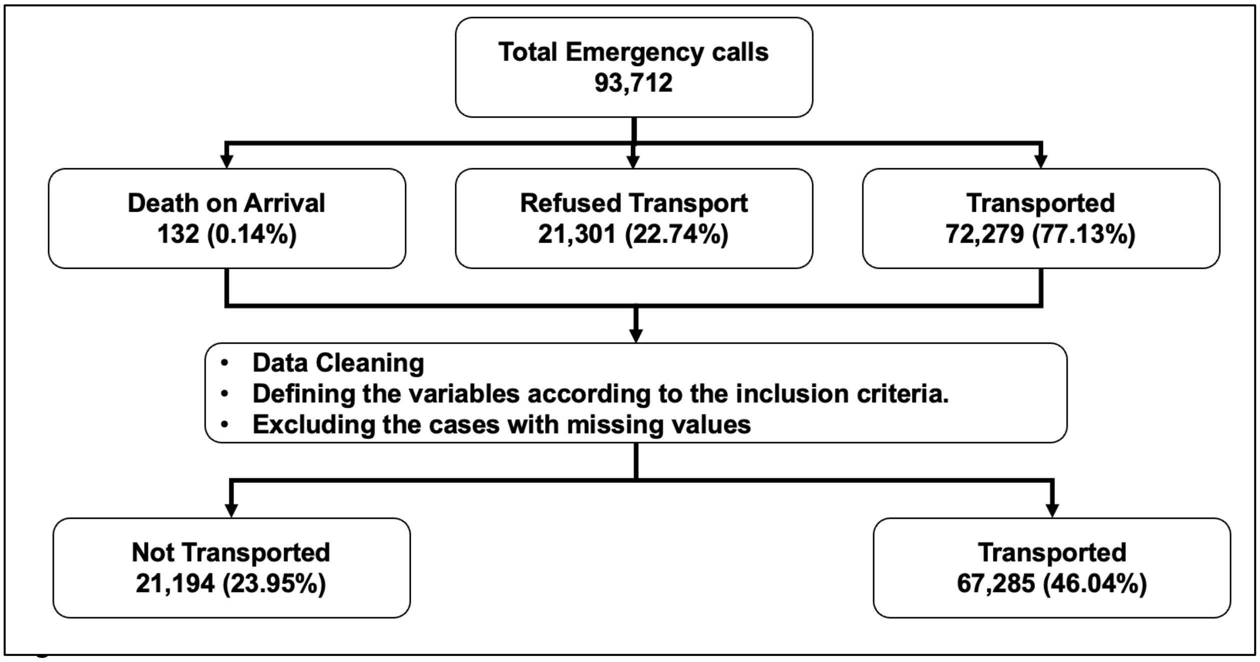

Appendix 2: Generalised Variance inflation factor analysis results

F **Results**			
**Variables**	GVIF	Df	GVIF^(1/(2*Df))
**CFS_Owner**	10,936.29	3	4.71
**PriorityToScene**	1.13	2	1.03
**PriorityToHospital**	103.47	4	1.78
**WeekNumber**	9.13	13	1.08
**Unit_Type**	11,565.1898	8	1.79
**Gender**	1.30	2	1.06
**Nationality_CAT**	1.91	10	1.03
**Age_CAT**	9.67	7	1.17
**Weight_CAT**	6.38	5	1.20
**ProvisonalDiagnoses_CAT**	11.50	41	1.03
**TransportedTo**	2,447.80	6	1.91
**PatientTriagedArea**	5,654.35	7	1.85
**TimeWithPatientUntilAvailable**	1.19	1	1.08
**TimeToFindTheNearestUnit**	1.05	1	1.02
**TimeToReachOnScene**	1.07	1	1.03
**Hour_Received**	1.02	1	1.01

Appendix 3: Linear regression results

Characteristic	Beta	95% CI[1]	p-value
**CFS_Owner**			
**Civil Defense**	—	—	
**EMS**	-0.81	-1.7, 0.12	0.087
**Other**	-1.9	-2.9, -0.98	<0.001
**Missing**	11	-3.1, 25	0.13
**PriorityToScene**			
**P1**	—	—	
**P2**	-0.02	-0.25, 0.21	0.9
**Missing**	-1.9	-2.9, -0.99	<0.001
**Unit_Type**			
**Alpha**	—	—	
**Bravo**	-4.0	-4.5, -3.4	<0.001
**Charlie**	-6.1	-6.5, -5.8	<0.001
**Delta**	-3.8	-4.1, -3.4	<0.001
**HAZMAT**	-4.4	-5.0, -3.8	<0.001
**LF**	-5.9	-6.9, -4.9	<0.001
**MIR**	-5.4	-6.0, -4.7	<0.001
**Missing**	-14	-28, 0.06	0.051
**Other**	-2.2	-2.7, -1.7	<0.001
**Gender**			
**Female**	—	—	
**Male**	-0.21	-0.40, -0.02	0.027
**Missing**	2.8	-2.0, 7.5	0.3
**Nationality_CAT**			
**East Asia & Pacific**	—	—	

**Europe & Central Asia**	0.32	-0.28, 0.92	0.3
**GCC Other**	0.33	-0.21, 0.88	0.2
**Latin America & Caribbean**	1.5	-0.62, 3.6	0.2
**MENA**	-0.01	-0.41, 0.39	>0.9
**Missing**	-0.68	-1.4, 0.01	0.053
**North America**	0.72	-0.56, 2.0	0.3
**Other**	-0.07	-0.79, 0.64	0.8
**Qatar**	0.09	-0.32, 0.50	0.7
**South Asia**	-0.24	-0.63, 0.15	0.2
**Sub-Saharan Africa**	-0.45	-0.89, -0.01	0.047
**Age_CAT**			
**14≤Age<29**	—	—	
**29≤Age<44**	0.25	0.04, 0.46	0.022
**44≤Age<59**	0.29	0.02, 0.57	0.039
**59≤Age<75**	0.49	0.14, 0.84	0.006
**75≤Age<90**	0.44	-0.05, 0.92	0.077
**Age<14**	-0.64	-1.3, -0.03	0.041
**Age≥90**	1.1	0.01, 2.3	0.049
**Missing**	0.09	-1.9, 2.1	>0.9
**Weight_CAT**			
**45≤Weight<70**	—	—	
**70≤Weight<95**	-0.03	-0.22, 0.15	0.7
**95≤Weight<120**	0.17	-0.20, 0.54	0.4
**Weight<45**	0.43	-0.16, 1.0	0.2
**Weight≥120**	1.2	0.32, 2.1	0.008
**Missing**	3.2	-0.17, 6.6	0.063
**ProvisonalDiagnoses_CAT**			
**Acute trauma**	—	—	

**Allergy/Anaphylaxis**	0.40	-0.45, 1.3	0.4
**Asthma**	0.55	-0.20, 1.3	0.15
**Burn**	0.01	-1.0, 1.0	>0.9
**Cardiac Arrest**	-0.40	-1.4, 0.58	0.4
**Cardiovas_ACS**	-0.14	-0.78, 0.49	0.7
**Cardiovas_Other**	7.0	-9.9, 24	0.4
**Cardiovas_SupVent**	0.76	-0.44, 2.0	0.2
**Cardiovas_Vent**	4.6	-0.85, 10	0.10
**Chronic Condition**	0.39	-0.22, 1.0	0.2
**COPD**	-0.63	-3.3, 2.0	0.6
**COVID19_Related**	2.3	1.3, 3.2	<0.001
**Croup/Epiglottitis**	2.7	0.50, 5.0	0.016
**CVA/TIA**	-1.1	-2.2, 0.14	0.083
**DOA**	-0.28	-2.1, 1.5	0.8
**Electrocution**	-2.1	-7.9, 3.7	0.5
**Endocrinology_Other**	-1.2	-11, 8.6	0.8
**Envenomation**	0.06	-4.0, 4.1	>0.9
**FBAO**	0.88	-1.8, 3.6	0.5
**Fever**	0.56	0.12, 1.0	0.013
**GI**	0.43	-0.03, 0.89	0.064
**GU**	-0.82	-2.0, 0.31	0.2
**Heat_related**	1.3	-1.8, 4.5	0.4
**Hemothorax**	-6.2	-13, 0.41	0.066
**Hyperglycemia**	-0.92	-1.9, 0.09	0.075
**Hypertension**	-0.08	-1.1, 0.95	0.9
**Hypoglycemia**	-0.44	-1.6, 0.74	0.5
**Low Acuity Problem_Medical**	0.31	-0.03, 0.66	0.078
**Low Acuity Problem_Trauma**	-0.52	-0.89, -0.15	0.006

**Near drowining**	3.8	-10, 18	0.6
**Neurology_Other**	0.36	-0.16, 0.88	0.2
**OBS_GYN**	-0.93	-1.8, -0.06	0.036
**Other**	0.08	-0.46, 0.61	0.8
**Pneumothorax**	9.1	-1.6, 20	0.10
**Respiratory_Infection**	0.58	0.03, 1.1	0.039
**Respiratory_Other**	0.03	-0.87, 0.93	>0.9
**Seizure**	-0.19	-0.89, 0.50	0.6
**Shock**	-2.4	-5.3, 0.61	0.12
**Syncope**	0.27	-0.65, 1.2	0.6
**Toxicology**	-0.05	-1.0, 0.93	>0.9
**Transport**	0.56	-1.1, 2.2	0.5
**Missing**	0.34	-3.6, 4.3	0.9
**TransportedTo**			
**Airport clinics**	—	—	
**Gouvernmental no Prenotif**	-1.1	-1.8, -0.44	0.001
**Gouvernmental with Prenotif**	-0.69	-2.4, 1.1	0.4
**Not Applicable**	0.55	-1.3, 2.4	0.6
**Other**	-0.92	-3.6, 1.7	0.5
**PEC**	-1.0	-2.5, 0.49	0.2
**Private**	-0.02	-1.3, 1.3	>0.9
**PatientTriagedArea**			
**Adult_Assessement_ED**	—	—	
**ByPass_Crit_ED**	1.4	0.90, 1.8	<0.001
**Dialysis**	-8.7	-26, 8.2	0.3
**Low_Acuity_ED**	-0.24	-0.47, 0.00	0.051
**Not Applicable**	-0.54	-2.3, 1.2	0.5
**Ob_Gyn_ED**	1.1	0.33, 1.9	0.005

**Other**	0.32	-0.64, 1.3	0.5
**Paed_ED**	-0.23	-1.6, 1.1	0.7
**TimeWithPatientUntilAvailable**	1.1	1.1, 1.1	<0.001
**TimeToFindTheNearestUnit**	-0.19	-0.26, -0.12	<0.001
**TimeToReachOnScene**	0.81	0.80, 0.83	<0.001
**Hour_Received**	-0.03	-0.05, -0.02	<0.001

[1] **CI = Confidence Interval**

1

Appendix 4: Cox Model Interaction Analysis Results

	coef	exp(coef)	se(coef)	97.50%
**Owner_UnitTypeCivil Defense_Charlie**	2.403609e+00 1	1.063028	1.16E+00	3.20E+00
**Owner_UnitTypeCivil Defense_Delta**	1.74E+00	5.708522	1.19E+00	2.44E+00
**Owner_UnitTypeCivil Defense_HAZMAT**	4.430416e+00 8	3.966373	1.27E+00	7.13E+00
**Owner_UnitTypeCivil Defense_LF**	2.29E+00	9.921681	1.45E+00	4.72E+00
**Owner_UnitTypeCivil Defense_MIR**	8.73E-01	2.394886	1.22E+00	1.29E+00
**Owner_UnitTypeCivil Defense_Other**	1.85E+00	6.338725	1.13E+00	2.35E+00
**Owner_UnitTypeEMS_Alpha**	2.13E+00	8.445045	1.11E+00	2.61E+00
**Owner_UnitTypeEMS_Bravo**	2.17E+00	8.756203	1.12E+00	2.70E+00
**Owner_UnitTypeEMS_Charlie**	3.276833e+00 2	6.491743	1.11E+00	4.02E+00
**Owner_UnitTypeEMS_Delta**	3.450040e+00 3	1.501664	1.11E+00	4.23E+00
**Owner_UnitTypeEMS_HAZMAT**	3.392513e+00 2	9.740589	1.11E+00	4.18E+00
**Owner_UnitTypeEMS_LF**	2.966759e+00 1	9.428856	1.13E+00	3.80E+00
**Owner_UnitTypeEMS_MIR**	3.499597e+00 3	3.102102	1.11E+00	4.32E+00
**Owner_UnitTypeEMS_Missing**	6.67E-05	1.000067	7.73E+144	6.30E+279
**Owner_UnitTypeEMS_Other**	3.409566e+00 3	0.252119	1.11E+00	4.20E+00
**Owner_UnitTypeMissing_Missing**	4.25E+04	Inf	1.71E+99	1.33E+199
**Owner_UnitTypeOther_Alpha**	1.71E+00	5.508328	1.11E+00	2.10E+00
**Owner_UnitTypeOther_Bravo**	2.07E+00	7.911508	1.48E+00	4.46E+00

**Owner_UnitTypeOther_Charlie**	2.442825e+00 1	1.505499	1.11E+00	3.02E+00
**Owner_UnitTypeOther_Delta**	2.386258e+00 1	0.872734	1.11E+00	2.94E+00
**Owner_UnitTypeOther_HAZMAT**	2.780365e+00 1	6.1249	1.16E+00	3.74E+00
**Owner_UnitTypeOther_LF**	2.984172e+00 1	9.770127	1.13E+00	3.76E+00
**Owner_UnitTypeOther_MIR**	2.492128e+00 1	2.086964	1.15E+00	3.28E+00
**Owner_UnitTypeOther_Missing**	3.64E-04	1.000364	2.42E+41	4.69E+77
**Owner_UnitTypeOther_Other**	2.330788e+00 1	0.286042	1.12E+00	2.90E+00
**ProtocolNameAllergies (Reactions) / Envenomations (Stings/ Bites)**	9.76E-01	2.654235	1.06E+00	1.10E+00
**ProtocolNameAnimal Bites / Attacks**	1.29E+00	3.633854	1.15E+00	1.69E+00
**ProtocolNameAssault**	9.25E-01	2.521988	1.05E+00	1.02E+00
**ProtocolNameBack Pain (Not Traumatic or Non-Recent Trauma)**	1.00E+00	2.723011	1.04E+00	1.08E+00
**ProtocolNameBreathing Problems**	9.63E-01	2.619851	1.03E+00	1.02E+00
**ProtocolNameBurns (Scalds) / Explosion (Blast)**	1.32E+00	3.751763	1.09E+00	1.56E+00
**ProtocolNameCarbon Monoxide / Inhalation / HAZMAT / CBRN**	5.02E-01	1.651392	1.14E+00	6.54E-01
**ProtocolNameCardiac or Respiratory Arrest / Death**	1.03E+00	2.8039	1.06E+00	1.16E+00
**ProtocolNameChest Pain / Chest Discomfort (Not Traumatic)**	1.04E+00	2.83258	1.03E+00	1.11E+00
**ProtocolNameChoking**	1.02E+00	2.768023	1.10E+00	1.22E+00
**ProtocolNameConvulsions / Seizures**	1.02E+00	2.767765	1.05E+00	1.11E+00
**ProtocolNameCriminal Incidents**	8.45E-01	2.327062	1.14E+00	1.09E+00
**ProtocolNameDiabetic Problems**	8.47E-01	2.332664	1.06E+00	9.53E-01

**ProtocolNameDrowning / Near Drowning / Diving / SCUBA Accident**	1.70E+00	5.491166	1.46E+00	3.59E+00
**ProtocolNameElectrocution / Lightning**	9.13E-01	2.491696	1.27E+00	1.45E+00
**ProtocolNameEye Problems / Injuries**	1.04E+00	2.835364	1.10E+00	1.25E+00
**ProtocolNameFalls**	1.14E+00	3.113583	1.03E+00	1.21E+00
**ProtocolNameFire Accidents**	1.25E+00	3.483975	1.41E+00	2.44E+00
**ProtocolNameHeadache**	9.84E-01	2.674347	1.05E+00	1.07E+00
**ProtocolNameHeart Problems / A.I.C.D.**	8.32E-01	2.297866	1.05E+00	9.15E-01
**ProtocolNameHemorrhage / Lacerations**	1.17E+00	3.210819	1.04E+00	1.27E+00
**ProtocolNameInaccessible Incident / Other Entrapments (Non-Traffic)**	7.37E-01	2.088699	1.12E+00	9.17E-01
**ProtocolNameIncidents of Rescue and Relief**	4.247511e+00 6	9.931105	1.52E+00	9.66E+00
**ProtocolNameMaritime Accidents**	2.492919e+00 1	2.096538	2.72E+00	1.77E+01
**ProtocolNameOverdose / Poisoning (Ingestion)**	9.35E-01	2.547552	1.09E+00	1.12E+00
**ProtocolNamePregnancy / Childbirth / Miscarriage**	1.13E+00	3.08065	1.05E+00	1.23E+00
**ProtocolNamePROQA**	1.69E+04	Inf	1.71E+99	5.29E+198
**ProtocolNamePsychiatric / Abnormal Behavior / Suicide Attempt**	1.11E+00	3.047222	1.05E+00	1.23E+00
**ProtocolNameSick Person (Specific Diagnosis)**	9.65E-01	2.62395	1.03E+00	1.02E+00
**ProtocolNameSpecial Services**	5.47E-01	1.728853	2.72E+00	3.90E+00
**ProtocolNameStab / Gunshot / Penetrating Trauma**	4.75E-01	1.607852	1.13E+00	6.05E-01
**ProtocolNameStroke (CVA) / Transient Ischemic Attack (TIA)**	1.07E+00	2.906699	1.07E+00	1.23E+00
**ProtocolNameTraffic / Transportation Incidents**	1.30E+00	3.658343	1.04E+00	1.39E+00

**ProtocolNameTransfer / Interfacility / Palliative Care**	9.63E-01	2.620708	1.06E+00	1.08E+00
**ProtocolNameTraumatic Injuries (Specific)**	1.14E+00	3.136692	1.04E+00	1.22E+00
**ProtocolNameUnconscious / Fainting (Near)**	1.02E+00	2.775275	1.03E+00	1.08E+00
**ProtocolNameUnknown Problem (Person Down)**	1.20E+00	3.313354	1.08E+00	1.39E+00
**ProtocolNameMissing**	NA	NA	1.00E+00	NA
**DispatchTypeT**	2.47E+04	Inf	1.71E+99	7.73E+198
**DispatchTypeUncompletedProQA**	NA	NA	1.00E+00	NA
**DispatchTypeX**	1.73E+04	Inf	1.71E+99	5.40E+198
**DispatchTypeY**	1.70E+04	Inf	1.71E+99	5.30E+198
**DispatchTypeZ**	1.76E+04	Inf	1.71E+99	5.50E+198
**DispatchTypeMissing**	NA	NA	1.00E+00	NA
**PriorityToSceneP2**	9.83E-01	2.672921	1.01E+00	1.01E+00
**PriorityToSceneMissing**	7.82E-01	2.185249	1.06E+00	8.75E-01
**RegionUnknown**	1.37E+00	3.934339	1.22E+00	2.03E+00
**RegionUrban**	1.04E+00	2.818891	1.01E+00	1.06E+00
**RegionMissing**	NA	NA	1.00E+00	NA
**LocationTypeBeach/Sea/Ocean**	1.45E+00	4.280818	1.07E+00	1.65E+00
**LocationTypeFarm**	1.33E+00	3.762865	1.08E+00	1.53E+00
**LocationTypeHome**	1.55E+00	4.729795	1.03E+00	1.63E+00
**LocationTypeIndustrial Area**	1.73E+00	5.618262	1.04E+00	1.85E+00

**LocationTypeOther**	1.25E+00	3.491735	1.03E+00	1.33E+00
**LocationTypePublic Area**	1.37E+00	3.929902	1.03E+00	1.46E+00
**LocationTypeRecreation (Sport)**	1.52E+00	4.581306	1.07E+00	1.74E+00
**LocationTypeSchool**	1.61E+00	5.012924	1.04E+00	1.74E+00
**LocationTypeStreet (Road)**	1.64E+00	5.137556	1.03E+00	1.73E+00
**LocationTypeWork**	1.75E+00	5.756851	1.03E+00	1.86E+00
**LocationTypeMissing**	1.27E+00	3.551248	1.04E+00	1.36E+00
**GenderMale**	1.02E+00	2.769922	1.02E+00	1.05E+00
**GenderMissing**	4.67E-01	1.595035	2.03E+00	1.88E+00
**Nationality_CATEurope & Central Asia**	7.94E-01	2.212657	1.03E+00	8.44E-01
**Nationality_CATGCC Other**	8.99E-01	2.456634	1.03E+00	9.47E-01
**Nationality_CATLatin America & Caribbean**	9.67E-01	2.629903	1.11E+00	1.19E+00
**Nationality_CATMENA**	9.13E-01	2.490955	1.02E+00	9.47E-01
**Nationality_CATMissing**	8.59E-01	2.361911	1.03E+00	9.17E-01
**Nationality_CATNorth America**	9.55E-01	2.599463	1.07E+00	1.09E+00
**Nationality_CATOther**	9.31E-01	2.535924	1.04E+00	9.98E-01
**Nationality_CATQatar**	8.34E-01	2.30145	1.02E+00	8.67E-01
**Nationality_CATSouth Asia**	1.01E+00	2.738985	1.02E+00	1.05E+00
**Nationality_CATSub-Saharan Africa**	1.01E+00	2.740803	1.02E+00	1.05E+00
**Age_CAT29≤Age<44**	9.62E-01	2.617585	1.02E+00	9.96E-01

**Age_CAT44≤Age<59**	1.03E+00	2.790178	1.02E+00	1.08E+00
**Age_CAT59≤Age<75**	1.00E+00	2.727899	1.03E+00	1.06E+00
**Age_CAT75≤Age<90**	9.18E-01	2.504219	1.03E+00	9.80E-01
**Age_CATAge<14**	1.19E+00	3.297687	1.03E+00	1.27E+00
**Age_CATAge≥90**	8.02E-01	2.229562	1.07E+00	9.23E-01
**Age_CATMissing**	8.31E-01	2.295486	1.21E+00	1.20E+00
**Weight_CAT70≤Weight<95**	9.96E-01	2.706577	1.01E+00	1.01E+00
**Weight_CAT95≤Weight<120**	1.00E+00	2.72403	1.02E+00	1.04E+00
**Weight_CATWeight<45**	9.51E-01	2.587044	1.03E+00	1.01E+00
**Weight_CATWeight≥120**	8.94E-01	2.444107	1.04E+00	9.70E-01
**Weight_CATMissing**	1.62E+00	5.078319	1.16E+00	2.18E+00
**ProvisonalDiagnoses_CATAllergy/Anaphylaxis**	1.11E+00	3.020115	1.05E+00	1.21E+00
**ProvisonalDiagnoses_CATAsthma**	7.65E-01	2.14851	1.04E+00	8.24E-01
**ProvisonalDiagnoses_CATBurn**	8.64E-01	2.373601	1.06E+00	9.69E-01
**ProvisonalDiagnoses_CATCardiac Arrest**	9.43E-01	2.566941	1.05E+00	1.04E+00
**ProvisonalDiagnoses_CATCardiovas_ACS**	1.06E+00	2.884027	1.03E+00	1.12E+00
**ProvisonalDiagnoses_CATCardiovas_Other**	1.27E+00	3.544078	2.03E+00	5.06E+00
**ProvisonalDiagnoses_CATCardiovas_SupVent**	1.20E+00	3.329108	1.06E+00	1.34E+00
**ProvisonalDiagnoses_CATCardiovas_Vent**	8.14E-01	2.256839	1.27E+00	1.29E+00
**ProvisonalDiagnoses_CATChronic Condition**	1.01E+00	2.741017	1.03E+00	1.07E+00

**ProvisonalDiagnoses_CATCOPD**	6.97E-01	2.007029	1.13E+00	8.79E-01
**ProvisonalDiagnoses_CATCOVID19_Related**	8.55E-01	2.352245	1.05E+00	9.34E-01
**ProvisonalDiagnoses_CATCroup/Epiglottitis**	1.15E+00	3.167215	1.10E+00	1.39E+00
**ProvisonalDiagnoses_CATCVA/TIA**	1.15E+00	3.148565	1.05E+00	1.27E+00
**ProvisonalDiagnoses_CATDOA**	1.28E+00	3.581563	1.08E+00	1.48E+00
**ProvisonalDiagnoses_CATElectrocution**	1.31E+00	3.72101	1.32E+00	2.28E+00
**ProvisonalDiagnoses_CATEndocrinology_Other**	1.69E+00	5.396892	1.51E+00	3.76E+00
**ProvisonalDiagnoses_CATEnvenomation**	9.92E-01	2.697761	1.21E+00	1.43E+00
**ProvisonalDiagnoses_CATFBAO**	9.32E-01	2.540706	1.13E+00	1.19E+00
**ProvisonalDiagnoses_CATFever**	8.57E-01	2.356291	1.02E+00	8.95E-01
**ProvisonalDiagnoses_CATGI**	9.71E-01	2.640762	1.02E+00	1.02E+00
**ProvisonalDiagnoses_CATGU**	1.25E+00	3.480404	1.05E+00	1.38E+00
**ProvisonalDiagnoses_CATHeat_related**	8.37E-01	2.308577	1.15E+00	1.10E+00
**ProvisonalDiagnoses_CATHemothorax**	1.60E+00	4.968168	1.32E+00	2.78E+00
**ProvisonalDiagnoses_CATHyperglycemia**	9.53E-01	2.592209	1.05E+00	1.05E+00
**ProvisonalDiagnoses_CATHypertension**	1.12E+00	3.073688	1.05E+00	1.23E+00
**ProvisonalDiagnoses_CATHypoglycemia**	6.08E-01	1.836155	1.07E+00	7.00E-01
**ProvisonalDiagnoses_CATLow Acuity Problem_Medical**	7.77E-01	2.175851	1.02E+00	8.04E-01
**ProvisonalDiagnoses_CATLow Acuity Problem_Trauma**	9.51E-01	2.589319	1.02E+00	9.84E-01
**ProvisonalDiagnoses_CATNear drowining**	8.60E-01	2.363255	2.03E+00	3.45E+00

<table>
<tr><td>ProvisonalDiagnoses_CATNeurology_Other</td><td>7.64E-01</td><td>2.146676</td><td>1.03E+00</td><td>8.07E-01</td></tr>
<tr><td>ProvisonalDiagnoses_CATOBS_GYN</td><td>1.24E+00</td><td>3.441425</td><td>1.04E+00</td><td>1.32E+00</td></tr>
<tr><td>ProvisonalDiagnoses_CATOther</td><td>9.95E-01</td><td>2.704863</td><td>1.03E+00</td><td>1.05E+00</td></tr>
<tr><td>ProvisonalDiagnoses_CATPneumothorax</td><td>7.14E-01</td><td>2.041802</td><td>1.56E+00</td><td>1.72E+00</td></tr>
<tr><td></td><td>z</td><td>Pr(>|z|)</td><td>2.50%</td><td></td></tr>
<tr><td>Owner_UnitTypeCivil Defense_Charlie</td><td>4.18E+02</td><td>1</td><td>1.81E+00</td><td></td></tr>
<tr><td>Owner_UnitTypeCivil Defense_Delta</td><td>2.49E+01</td><td>1.001299</td><td>1.24E+00</td><td></td></tr>
<tr><td>Owner_UnitTypeCivil Defense_HAZMAT</td><td>4.60E+02</td><td>1</td><td>2.75E+00</td><td></td></tr>
<tr><td>Owner_UnitTypeCivil Defense_LF</td><td>9.54E+00</td><td>1.024416</td><td>1.11E+00</td><td></td></tr>
<tr><td>Owner_UnitTypeCivil Defense_MIR</td><td>5.06E-01</td><td>1.642186</td><td>5.91E-01</td><td></td></tr>
<tr><td>Owner_UnitTypeCivil Defense_Other</td><td>1.47E+02</td><td>1.000001</td><td>1.45E+00</td><td></td></tr>
<tr><td>Owner_UnitTypeEMS_Alpha</td><td>1.60E+03</td><td>1</td><td>1.74E+00</td><td></td></tr>
<tr><td>Owner_UnitTypeEMS_Bravo</td><td>9.99E+02</td><td>1</td><td>1.74E+00</td><td></td></tr>
<tr><td>Owner_UnitTypeEMS_Charlie</td><td>9.08E+04</td><td>1</td><td>2.67E+00</td><td></td></tr>
<tr><td>Owner_UnitTypeEMS_Delta</td><td>1.41E+05</td><td>1</td><td>2.81E+00</td><td></td></tr>
<tr><td>Owner_UnitTypeEMS_HAZMAT</td><td>9.37E+04</td><td>1</td><td>2.75E+00</td><td></td></tr>
<tr><td>Owner_UnitTypeEMS_LF</td><td>5.54E+03</td><td>1</td><td>2.32E+00</td><td></td></tr>
<tr><td>Owner_UnitTypeEMS_MIR</td><td>1.19E+05</td><td>1</td><td>2.84E+00</td><td></td></tr>
<tr><td>Owner_UnitTypeEMS_Missing</td><td>9.72E-01</td><td>2.656493</td><td>7.06E-289</td><td></td></tr>
<tr><td>Owner_UnitTypeEMS_Other</td><td>9.97E+04</td><td>1</td><td>2.77E+00</td><td></td></tr>
</table>

**Owner_UnitTypeMissing_Missing**	1.05E+00	2.619007	1.36E-190
**Owner_UnitTypeOther_Alpha**	1.61E+02	1	1.39E+00
**Owner_UnitTypeOther_Bravo**	6.37E+00	1.066081	9.59E-01
**Owner_UnitTypeOther_Charlie**	3.77E+03	1	1.98E+00
**Owner_UnitTypeOther_Delta**	3.39E+03	1	1.93E+00
**Owner_UnitTypeOther_HAZMAT**	8.85E+02	1	2.07E+00
**Owner_UnitTypeOther_LF**	1.05E+04	1	2.37E+00
**Owner_UnitTypeOther_MIR**	6.63E+02	1	1.89E+00
**Owner_UnitTypeOther_Missing**	9.20E-01	2.544069	2.82E-85
**Owner_UnitTypeOther_Other**	2.08E+03	1	1.88E+00
**ProtocolNameAllergies (Reactions) / Envenomations (Stings/ Bites)**	6.75E-01	2.002017	8.66E-01
**ProtocolNameAnimal Bites / Attacks**	6.24E+00	1.069297	9.82E-01
**ProtocolNameAssault**	2.11E-01	1.127786	8.38E-01
**ProtocolNameBack Pain (Non-Traumatic or Non-Recent Trauma)**	1.05E+00	2.615972	9.33E-01
**ProtocolNameBreathing Problems**	2.66E-01	1.203609	9.11E-01
**ProtocolNameBurns (Scalds) / Explosion (Blast)**	2.82E+01	1.000843	1.12E+00
**ProtocolNameCarbon Monoxide / Inhalation / HAZMAT / CBRN**	6.05E-03	1	3.85E-01
**ProtocolNameCardiac or Respiratory Arrest / Death**	1.63E+00	1.865362	9.13E-01
**ProtocolNameChest Pain / Chest Discomfort (Non-Traumatic)**	3.76E+00	1.203778	9.81E-01
**ProtocolNameChoking**	1.21E+00	2.336236	8.47E-01

**ProtocolNameConvulsions / Seizures**	1.50E+00	1.988531	9.33E-01
**ProtocolNameCriminal Incidents**	2.67E-01	1.20493	6.57E-01
**ProtocolNameDiabetic Problems**	6.26E-02	1.005616	7.53E-01
**ProtocolNameDrowning / Near Drowning / Diving / SCUBA Accident**	4.05E+00	1.17564	8.08E-01
**ProtocolNameElectrocution / Lightning**	6.82E-01	2.016742	5.73E-01
**ProtocolNameEye Problems / Injuries**	1.56E+00	1.930272	8.68E-01
**ProtocolNameFalls**	5.02E+01	1.00009	1.07E+00
**ProtocolNameFire Accidents**	1.91E+00	1.677717	6.38E-01
**ProtocolNameHeadache**	6.94E-01	2.044707	9.01E-01
**ProtocolNameHeart Problems / A.I.C.D.**	2.28E-02	1.000157	7.56E-01
**ProtocolNameHemorrhage / Lacerations**	3.59E+01	1.000344	1.07E+00
**ProtocolNameInaccessible Incident / Other Entrapments (Non-Traffic)**	6.50E-02	1.0063	5.91E-01
**ProtocolNameIncidents of Rescue and Relief**	3.16E+01	1.000557	1.87E+00
**ProtocolNameMaritime Accidents**	2.49E+00	1.43553	3.50E-01
**ProtocolNameOverdose / Poisoning (Ingestion)**	4.74E-01	1.577488	7.84E-01
**ProtocolNamePregnancy / Childbirth / Miscarriage**	1.37E+01	1.008974	1.03E+00
**ProtocolNamePROQA**	1.04E+00	2.627452	5.40E-191
**ProtocolNamePsychiatric / Abnormal Behavior / Suicide Attempt**	8.84E+00	1.029785	1.01E+00
**ProtocolNameSick Person (Specific Diagnosis)**	2.70E-01	1.209295	9.14E-01
**ProtocolNameSpecial Services**	5.48E-01	1.729198	7.68E-02

**ProtocolNameStab / Gunshot / Penetrating Trauma**	2.37E-03	1	3.73E-01
**ProtocolNameStroke (CVA) / Transient Ischemic Attack (TIA)**	2.51E+00	1.431128	9.29E-01
**ProtocolNameTraffic / Transportation Incidents**	1.17E+03	1	1.21E+00
**ProtocolNameTransfer / Interfacility / Palliative Care**	5.28E-01	1.686513	8.59E-01
**ProtocolNameTraumatic Injuries (Specific)**	4.66E+01	1.000122	1.07E+00
**ProtocolNameUnconscious / Fainting (Near)**	1.99E+00	1.636129	9.63E-01
**ProtocolNameUnknown Problem (Person Down)**	1.03E+01	1.019878	1.03E+00
**ProtocolNameMissing**	NA	NA	NA
**DispatchTypeT**	1.05E+00	2.623976	7.90E-191
**DispatchTypeUncompletedProQA**	NA	NA	NA
**DispatchTypeX**	1.04E+00	2.627265	5.52E-191
**DispatchTypeY**	1.04E+00	2.627428	5.42E-191
**DispatchTypeZ**	1.04E+00	2.627097	5.62E-191
**DispatchTypeMissing**	NA	NA	NA
**PriorityToSceneP2**	2.88E-01	1.238358	9.57E-01
**PriorityToSceneMissing**	1.38E-02	1.000019	6.98E-01
**RegionUnknown**	4.84E+00	1.121887	9.26E-01
**RegionUrban**	2.32E+01	1.001671	1.01E+00
**RegionMissing**	NA	NA	NA
**LocationTypeBeach/Sea/Ocean**	3.06E+02	1	1.28E+00

**LocationTypeFarm**	4.38E+01	1.000157	1.15E+00
**LocationTypeHome**	2.65E+07	1	1.48E+00
**LocationTypeIndustrial Area**	3.74E+06	1	1.61E+00
**LocationTypeOther**	8.98E+02	1	1.17E+00
**LocationTypePublic Area**	1.25E+04	1	1.28E+00
**LocationTypeRecreation (Sport)**	4.69E+02	1	1.33E+00
**LocationTypeSchool**	2.12E+05	1	1.49E+00
**LocationTypeStreet (Road)**	5.02E+07	1	1.55E+00
**LocationTypeWork**	1.03E+08	1	1.65E+00
**LocationTypeMissing**	7.63E+02	1	1.18E+00
**GenderMale**	2.92E+00	1.328153	9.85E-01
**GenderMissing**	3.42E-01	1.327134	1.16E-01
**Nationality_CATEurope & Central Asia**	6.20E-04	1	7.47E-01
**Nationality_CATGCC Other**	1.84E-02	1.000065	8.53E-01
**Nationality_CATLatin America & Caribbean**	7.31E-01	2.12484	7.84E-01
**Nationality_CATMENA**	8.36E-03	1.000002	8.79E-01
**Nationality_CATMissing**	1.06E-02	1.000005	8.05E-01
**Nationality_CATNorth America**	5.04E-01	1.638213	8.38E-01
**Nationality_CATOther**	1.32E-01	1.044011	8.68E-01
**Nationality_CATQatar**	1.16E-04	1	8.01E-01

**Nationality_CATSouth Asia**	1.50E+00	1.983897	9.71E-01
**Nationality_CATSub-Saharan Africa**	1.48E+00	2.00839	9.67E-01
**Age_CAT29≤Age<44**	1.15E-01	1.030718	9.29E-01
**Age_CAT44≤Age<59**	2.95E+00	1.32217	9.79E-01
**Age_CAT59≤Age<75**	1.15E+00	2.440486	9.54E-01
**Age_CAT75≤Age<90**	7.66E-02	1.01024	8.60E-01
**Age_CATAge<14**	2.05E+02	1	1.12E+00
**Age_CATAge≥90**	4.63E-02	1.00213	6.96E-01
**Age_CATMissing**	3.72E-01	1.380456	5.76E-01
**Weight_CAT70≤Weight<95**	6.23E-01	1.888639	9.78E-01
**Weight_CAT95≤Weight<120**	1.13E+00	2.472054	9.68E-01
**Weight_CATWeight<45**	1.73E-01	1.082382	8.98E-01
**Weight_CATWeight≥120**	6.71E-02	1.006919	8.24E-01
**Weight_CATMissing**	2.60E+01	1.001125	1.21E+00
**ProvisonalDiagnoses_CATAllergy/Anaphylaxis**	8.04E+00	1.03781	1.01E+00
**ProvisonalDiagnoses_CATAsthma**	8.44E-04	1	7.10E-01
**ProvisonalDiagnoses_CATBurn**	8.17E-02	1.012332	7.71E-01
**ProvisonalDiagnoses_CATCardiac Arrest**	2.97E-01	1.252231	8.57E-01
**ProvisonalDiagnoses_CATCardiovas_ACS**	6.82E+00	1.056506	9.99E-01
**ProvisonalDiagnoses_CATCardiovas_Other**	1.39E+00	2.094818	3.16E-01

**ProvisonalDiagnoses_CATCardiovas_SupVent**	3.11E+01	1.00059	1.08E+00
**ProvisonalDiagnoses_CATCardiovas_Vent**	4.19E-01	1.468938	5.12E-01
**ProvisonalDiagnoses_CATChronic Condition**	1.33E+00	2.168666	9.53E-01
**ProvisonalDiagnoses_CATCOPD**	4.75E-02	1.002321	5.52E-01
**ProvisonalDiagnoses_CATCOVID19_Related**	3.08E-02	1.000502	7.83E-01
**ProvisonalDiagnoses_CATCroup/Epiglottitis**	4.40E+00	1.14839	9.55E-01
**ProvisonalDiagnoses_CATCVA/TIA**	1.33E+01	1.009627	1.03E+00
**ProvisonalDiagnoses_CATDOA**	2.63E+01	1.001071	1.10E+00
**ProvisonalDiagnoses_CATElectrocution**	2.65E+00	1.390445	7.59E-01
**ProvisonalDiagnoses_CATEndocrinology_Other**	3.59E+00	1.22343	7.56E-01
**ProvisonalDiagnoses_CATEnvenomation**	9.60E-01	2.631594	6.88E-01
**ProvisonalDiagnoses_CATFBAO**	5.74E-01	1.784843	7.28E-01
**ProvisonalDiagnoses_CATFever**	9.78E-04	1	8.21E-01
**ProvisonalDiagnoses_CATGI**	2.74E-01	1.215747	9.29E-01
**ProvisonalDiagnoses_CATGU**	8.22E+01	1.00001	1.13E+00
**ProvisonalDiagnoses_CATHeat_related**	2.80E-01	1.225016	6.36E-01
**ProvisonalDiagnoses_CATHemothorax**	5.39E+00	1.096298	9.26E-01
**ProvisonalDiagnoses_CATHyperglycemia**	3.74E-01	1.384083	8.65E-01
**ProvisonalDiagnoses_CATHypertension**	1.24E+01	1.011985	1.03E+00
**ProvisonalDiagnoses_CATHypoglycemia**	1.01E-03	1	5.27E-01

**ProvisonalDiagnoses_CATLow Acuity Problem_Medical**	4.60E-07	1	7.52E-01
**ProvisonalDiagnoses_CATLow Acuity Problem_Trauma**	5.41E-02	1.003544	9.20E-01
**ProvisonalDiagnoses_CATNear drowining**	8.08E-01	2.297027	2.14E-01
**ProvisonalDiagnoses_CATNeurology_Other**	5.98E-05	1	7.24E-01
**ProvisonalDiagnoses_CATOBS_GYN**	3.91E+02	1	1.15E+00
**ProvisonalDiagnoses_CATOther**	8.20E-01	2.322876	9.47E-01
**ProvisonalDiagnoses_CATPneumothorax**	4.71E-01	1.570653	2.97E-01

Analysis of Deviance Table

Cox model: response is Surv(TimeFromDispatchUntilAvailable, Handover == 'Not Transported')

Terms added sequentially (first to last)

loglik Chisq Df Pr(>|Chi|)

NULL -226130

Transport_Group -189935 72390 1 < 2.2e-16 ***

Signif. codes: 0 '*' 0.001 '**' 0.01 '*' 0.05 '.' 0.1 ' ' 1**

Appendix 5: Cox model analysis of variance results for the Transport Group

ANOVA Result

Cox Model Analysis

Variable Name	Log-Likelihood	χ^2	Degrees of Freedom	P-value
**Owner_UnitType**	-689236.1	8012.17	18	<0.01
**ProtocolName**	-688492.7	1486.88	35	<0.01
**DispatchType**	-688467.7	49.92	5	<0.01
**PriorityToScene**	-688445.6	44.14	2	<0.01
**Region**	-688171.9	547.45	2	<0.01
**LocationType**	-687886.7	570.31	11	<0.01
**Gender**	-687863.4	46.62	2	<0.01
**Nationality_CAT**	-687678.8	369.19	10	<0.01
**Age_CAT**	-687617.5	122.71	7	<0.01
**Weight_CAT**	-687610.7	13.56	5	<0.01
**ProvisonalDiagnoses_CAT**	-687306.2	608.89	41	<0.01
**TimeWithPatientUntilAvailable**	-636964.2	100684.10	1	<0.01
**TimeToFindTheNearestUnit**	-636863.7	201.06	1	<0.01
**TimeToReachOnScene**	-630404.6	12918.20	1	<0.01
**Hour_Received**	-630403.9	1.33	1	0.24
**Asthma**	-630403.7	0.46	1	0.49
**CAD**	-630403.5	0.28	1	0.59
**COPD**	-630401.7	3.53	1	0.06
**CVA**	-630398.9	5.76	1	0.01
**Seizure**	-630396.1	5.46	1	0.02
**DM**	-630386.2	19.85	1	<0.01
**Hypertension**	-630381.2	10.01	1	<0.01

**Surgeries**	-630372.3	17.80	1	<0.01
**Currently Pregnant**	-630370.9	2.79	2	0.02

Analysis of Variance Table

Cox model: response is Surv(TimeFromDispatchUntilAvailable, Handover == 'Not Transported') Terms added sequentially (first to last)

**loglik**	Chisq	Df	Pr(>|Chi|)
**NULL-226130**			
**Transport_Group -189935**	72390	1	$< 2.2\times 10^{-16}$ ***

Signif. codes: 0 '*' 0.001 '**' 0.01 '*' 0.05 '.' 0.1 ' ' 1**

Appendix 5: Schoenfeld Analysis Plots

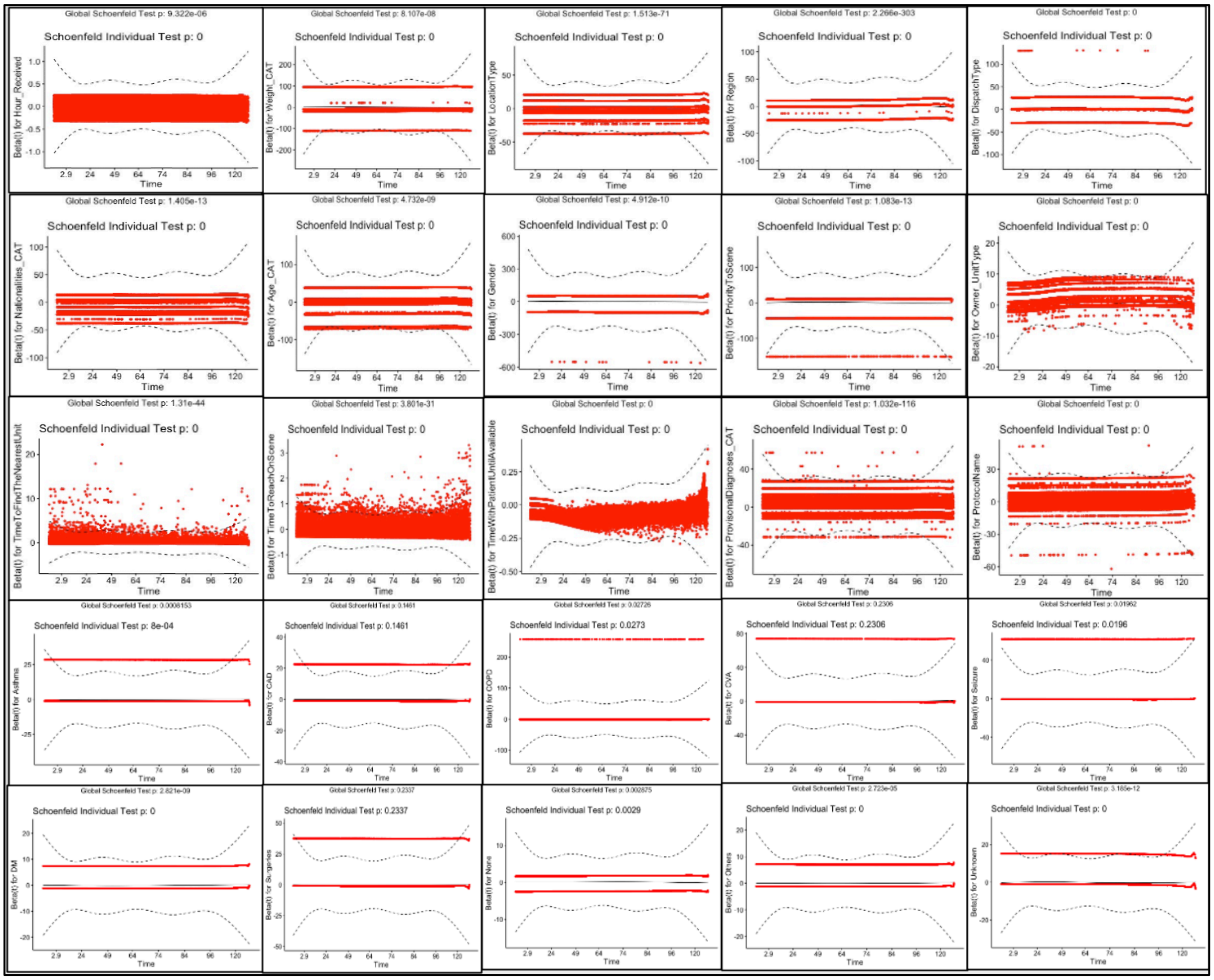

Appendix 6: Hazard Ratio Plots for Demographic Variables

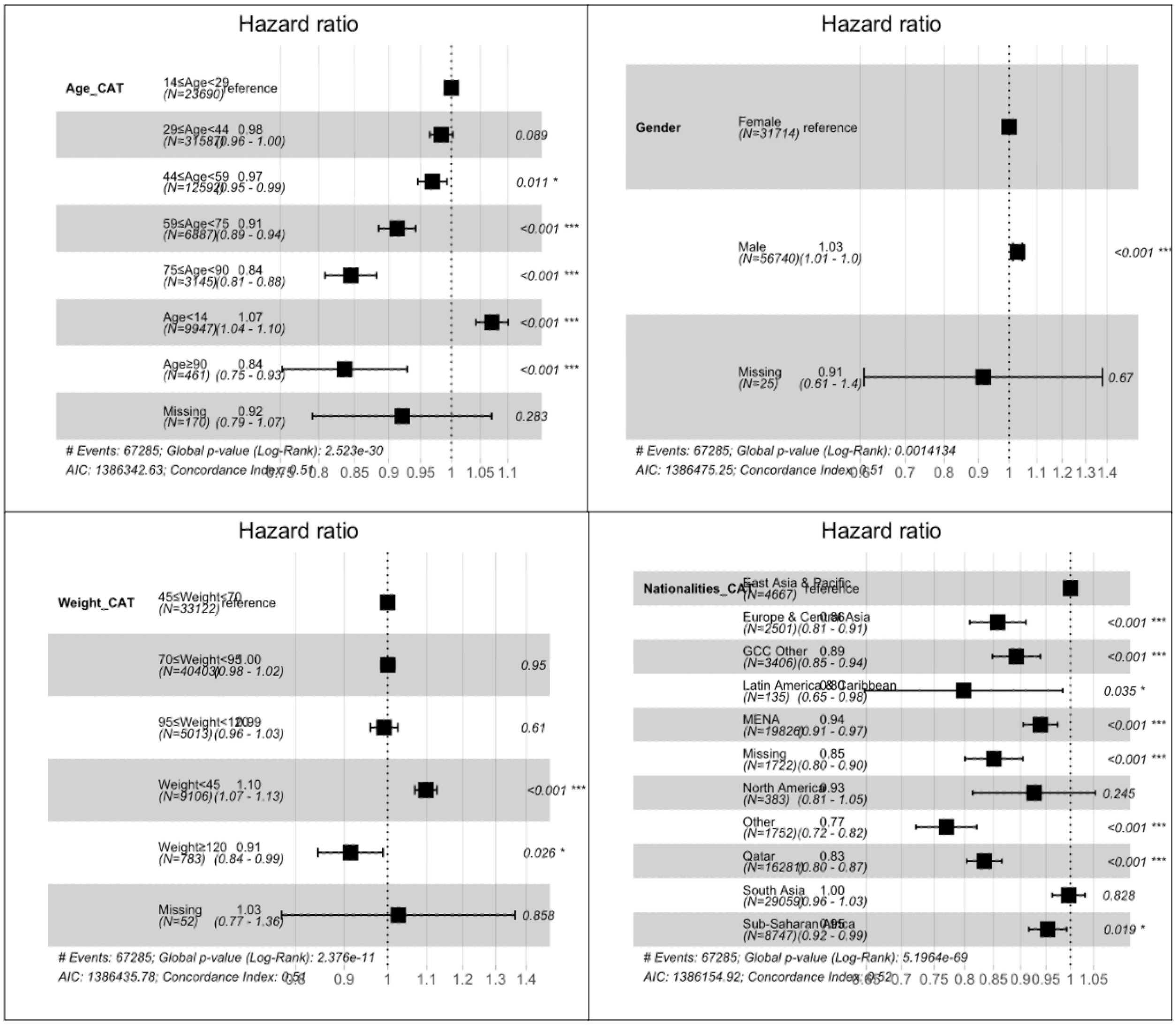

Appendix 7: The R-pseudo code used for the survival analysis

1. Data Preparation and Transformation

- Load Necessary Libraries
    - **survival**, **ggplot2**, **ggplotify**, **cmprsk**.
- Load Raw Data
    - Read raw data into a variable called **RawData**.
- Preprocess and Filter Data
    - Convert columns to appropriate formats.
    - Filter and subset the data as per the conditions provided in the code.
- Data Transformation
    - Melt the **RawData** to create **RawData_long**.
    - Filter the **RawData_long** to create **RawData_long_filtered**.

2. Kaplan-Meier Analysis

- Kaplan-Meier Curve Stratified by Gender, Nationalities, Age, and Weight
    - Perform Kaplan-Meier analysis on different strata.
    - Create Kaplan-Meier plots for different strata.
    - Arrange and display the plots using **grid.arrange**.
- Kaplan-Meier Curve Stratified by Top 3 Conditions
    - Identify the top 3 conditions.
    - Filter the data based on these conditions.
    - Perform Kaplan-Meier analysis and create the plot.
    - Add the plot to the arranged plots from previous steps.

3. Cox Proportional Hazards Model

- For each variable in **vars**:
    - Fit a Cox proportional hazards model.
    - Perform ANOVA on the model and print the results.
    - Check the proportional hazards assumption using Schoenfeld residuals.
    - Plot Schoenfeld residuals.
    - Also, make forest plots for each variable.

4. Competing Risk Analysis

- Define Events
    - Encode the different event types (Transported, Not Transported, No Event).
- Compute Cumulative Incidence Function (CIF)
    - Calculate CIF for competing risks using **cuminc**.
- Plot CIF
    - Plot the CIF for the defined events with appropriate labels and legend.
- Compute and Display Median Time to Event
    - Compute the median time to event or censoring for 'Not Transported' event.
    - Display the result.
